# Streamflow variations across the Andes (18°–55°S) during the instrumental era

**DOI:** 10.1038/s41598-019-53981-x

**Published:** 2019-11-29

**Authors:** M. H. Masiokas, L. Cara, R. Villalba, P. Pitte, B. H. Luckman, E. Toum, D. A. Christie, C. Le Quesne, S. Mauget

**Affiliations:** 1 Instituto Argentino de Nivología, Glaciología y Ciencias Ambientales (IANIGLA), CCT- CONICET Mendoza, C.C. 330, (5500) Mendoza, Argentina; 2 Department of Geography, University of Western Ontario, Ontario, Canada; 3 Laboratorio de Dendrocronología y Cambio Global, Instituto de Conservación Biodiversidad y Territorio, Facultad de Ciencias Forestales y Recursos Naturales, Universidad Austral de Chile, Valdivia, Chile; 4 Center for Climate and Resilience Research (CR)2, Santiago, Chile; 5 Wind Erosion and Water Conservation Unit, Agricultural Research Service, U.S. Department of Agriculture, Lubbock, Texas USA

**Keywords:** Hydrology, Hydrology

## Abstract

The rivers originating in the southern Andes (18°–55°S) support numerous ecosystems and a large number of human populations and socio-economic activities in the adjacent lowlands of Chile, Argentina and Bolivia. Here we show that ca. 75% of the total variance in the streamflow records from this extensive region can be explained by only eight spatially coherent patterns of variability. Five (three) of these Andean patterns exhibit extreme dry (wet) conditions in recent years, with strong interannual variations in northern Chile; long-term drying trends between 31° and 41°S; a transitional pattern in the central Patagonian Andes; and increasing trends in northwestern Argentina and southern Bolivia, the Fueguian Andes, and the eastern portion of the South Patagonian Icefield. Multivariate regression analyses show that large-scale indices of ENSO variability can predict 20% to 45% of annual runoff variability between 28° and 46°S. The influence of Antarctic and North Pacific indices becomes more relevant south of 43°S and in northwestern Argentina and southern Bolivia, respectively, but their overall skill as predictors of Andean streamflows is weak. The analyses provide relevant new information to improve understanding of the spatial coherence, the main temporal features, and the ocean-atmospheric forcings of surface runoff across the southern Andes.

## Introduction

The southern Andes, located along the western margin of South America between ca. 18° and 55°S, contain the upper portions of all of the most important river basins in Chile, western Argentina and southern Bolivia. More than 25 million people live in these basins and depend, directly or indirectly, on Andean water resources for human consumption, irrigation, industries, tourism, recreation, hydropower generation, and aquifer recharge. The rivers in this region also sustain an immense variety of ecosystems and are crucial for the preservation of biodiversity not only in the mountains but also on the adjacent lowland areas. However, despite many studies that have characterized the main hydrological features and trends in different sectors of the southern Andes, there has not been a large-scale assessment of the main spatio-temporal patterns of hydrological variations using *in situ* discharge measurements from this extensive and diverse region. In part, this is due to the large area occupied by this region (>50% of the total Andean range) and the fact that not all river records were publicly and readily available for analysis until recent times (see e.g.^1^).

**Figure 1:**
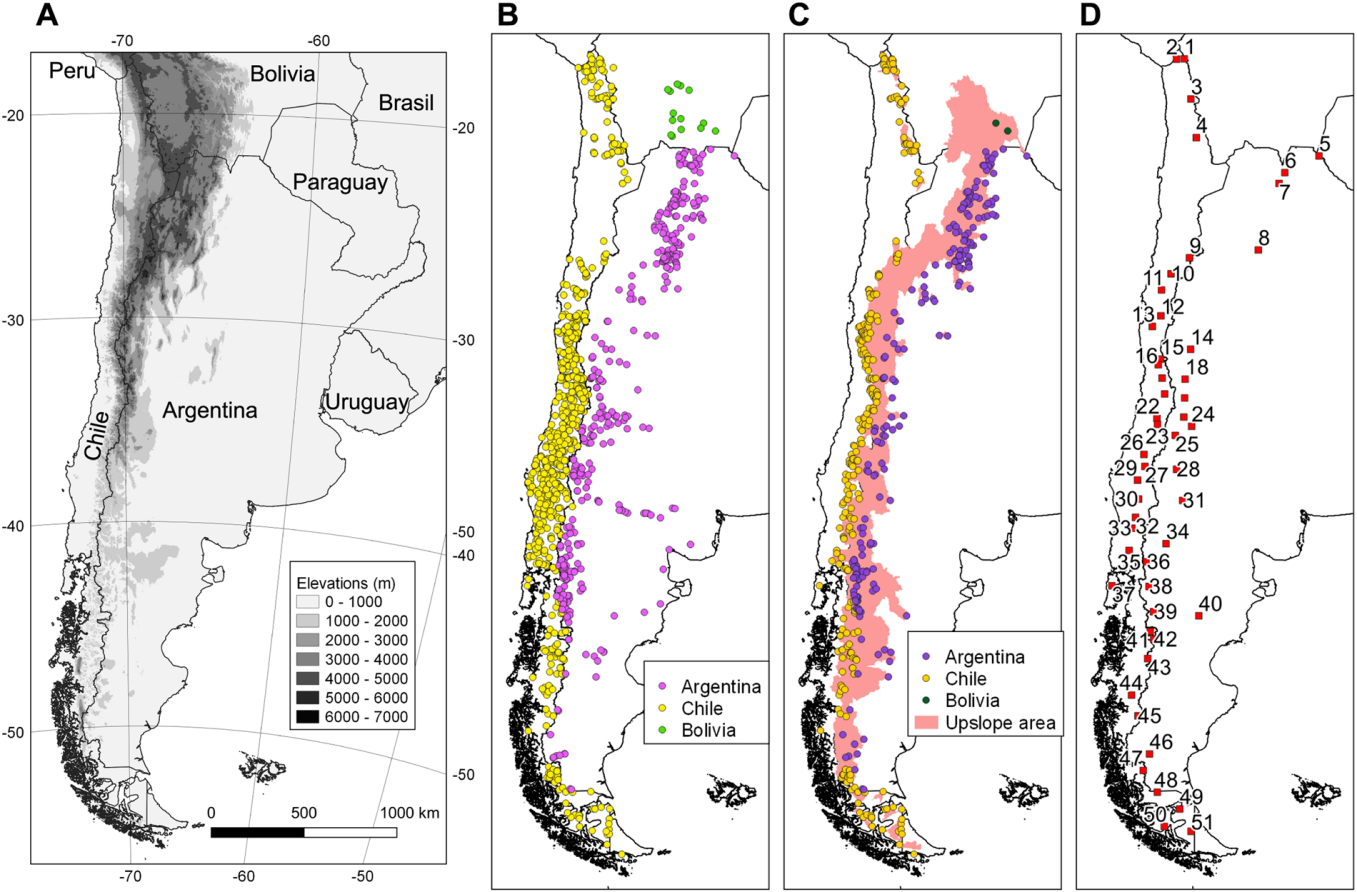
Elevation changes and available streamflow data in the southern Andes. **(A)** Variations in elevation of the southern Andes in Chile, western Argentina and south-western Bolivia. The black area in the southwestern sector reflects the complex detail of the coastline. (**B)** Location of all the streamflow stations available for this study (n = 1226). (**C)** Selected stations in Chile, Bolivia and Argentina (n = 513). The pink shading shows the upslope area aggregated for all these selected sites. (**D)** Location of the reference stations representing basins with long, complete river records (n = 51; see Supplementary Table 1). The records from these reference basins were used in the assessment of spatial and temporal patterns of variations in the southern Andes (see Data and Methods for details).

Here we identify and discuss the main modes of streamflow variability across the Andes between ca. 18° and 55°S using an integrated database of mean monthly surface runoff records from Chile, western Argentina and southern Bolivia (Fig. 1). This database is derived from official records collected by national and local water resource agencies, and contains some series which are over 100 years long (see Supplementary Table 1). This is particularly noteworthy because in most Andean river basins the length of the streamflow records far exceeds that of the available climate series, which are usually scarce, short and incomplete at high elevation sites (see e.g. refs. ^2,3^). In many of these basins, the streamflow records represent the only direct *in situ* measurements that can be used to detect and characterize recent hydro-climatic changes and evaluate their significance in a long term context. Considering that streamflow integrates, via the hydrological balance equation, the rainfall and evapotranspiration that occur over a given watershed, these runoff records can also provide valuable complementary information for many related studies (e.g. hydrological, ecological, etc.) in extensive and poorly known sectors of the Andes.

In this study we first use a set of statistical approaches to identify the main spatial and temporal patterns of variability in streamflow records from the southern Andes over the 1986–2015 period. We then develop regionally-averaged series of more extended duration using the longest and best correlated records from each sector, and evaluate the existence and relative magnitude of recent extreme dry or wet periods in each region. This particular set of techniques was considered an appropriate approach to handle the heterogeneous spatio-temporal coverage of available data and to enhance the common regional hydroclimatic signal while minimizing the impact of any undetected non-climatic inhomogeneities in the original series. Although by no means definitive, these analyses provide an original overview of the most important hydrological patterns of the southern Andes, and can help improve the current understanding and management of the water resources that originate in this mountain range. The integration of records from different sources into a single homogeneous database will hopefully facilitate a wide range of related studies, including those interested in the detection and characterization of the impacts of climate change in poorly known sectors of the Chilean, Argentinean and Bolivian Andes. We conclude the manuscript performing multiple regression analyses where various large-scale ocean-atmospheric indices from the Pacific, Atlantic and the Southern Ocean are used as potential predictors of the variations observed in the streamflow patterns of the southern Andes. Given the socio-economic importance of most of these Andean rivers, these results can provide relevant information for the development of reliable predictive models of surface runoff variability based on easily accessible atmospheric variables or climatic indices (see e.g. ref. ^4^).

## Results

As discussed in the Data and Methods section (see below), the processing of the original mean monthly streamflow records included their statistical aggregation into one reference series per river basin, and the subsequent application of Principal Component Analysis (PCA) to identify their main modes of variability over the 1986–2015 period (shared by the largest number of series). This procedure effectively reduced the number of records under investigation and allowed the identification of eight spatially consistent, major streamflow patterns across the southern Andes. These sub-regions show no spatial overlap and have distinctive temporal signatures that can help to improve the understanding of the hydro-climatic nature and the recent surface runoff variations of different sectors within the Andes south of 18°S.

### Identification of the main regional streamflow patterns

**Figure 2:**
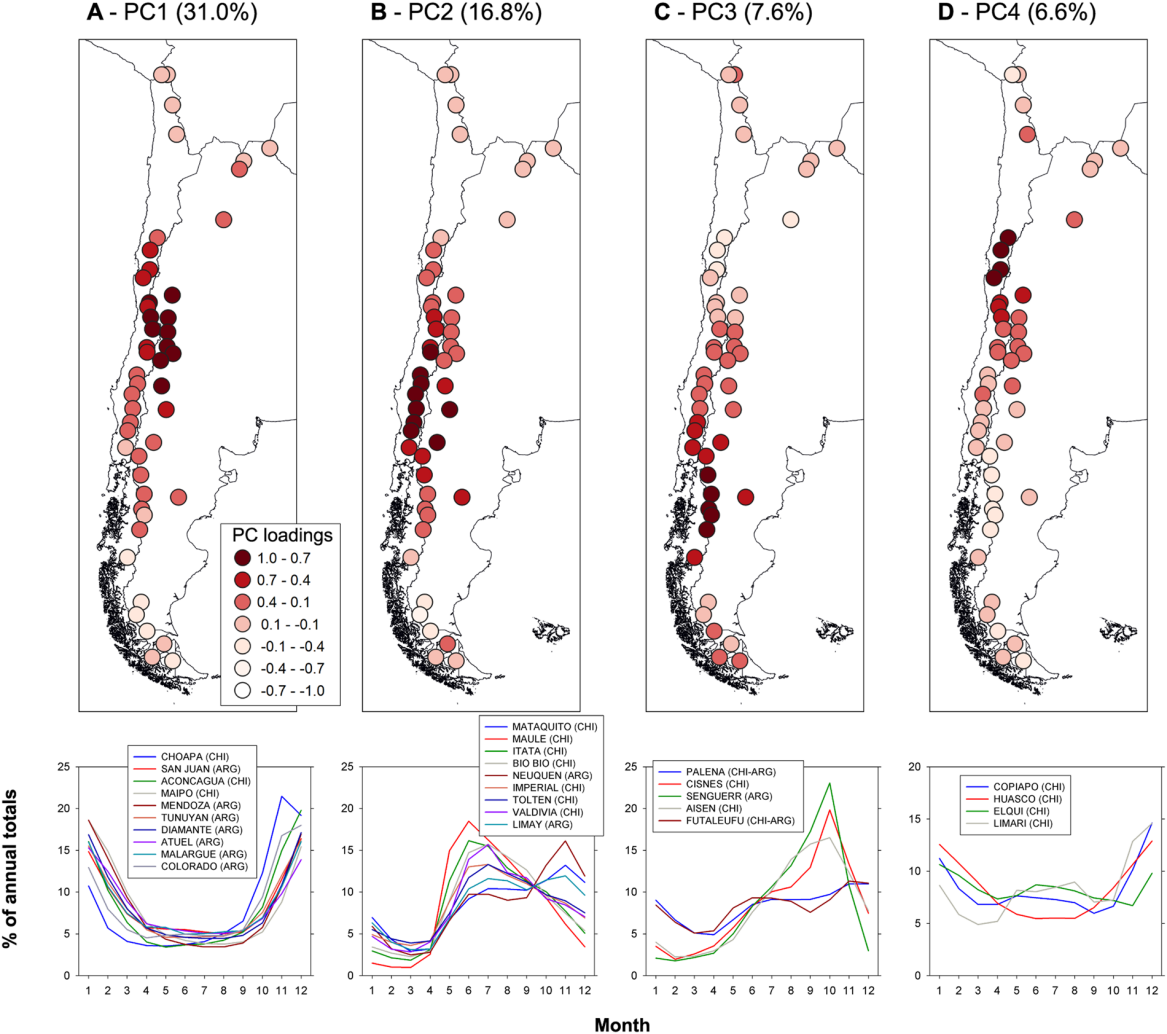
(**A)** Above: Map of the loadings of the first principal component (PC1) derived from 48 mean monthly streamflow series over the April 1986 – March 2016 period. The percentage of total variance accounted for by this component is shown in parentheses. The lower diagram portrays the monthly variations in surface runoff (expressed as percentages of the annual totals) for the stations with PC loadings >0.7 identified in the map above (i.e. those with dark red circles). The month 1 = January, 2 = February, 3 = March, etc. (**B–D)** Same as A, but for PC2, PC3, and PC4, respectively.

The first sub-region (PC1; Fig. 2A) explains the largest proportion of variance in the dataset (31.0%) and includes 10 major river basins along an extensive portion of the central Andes of Chile and Argentina. The list includes, for example, the Aconcagua and Maipo rivers, which provide freshwater for a large proportion of the human activities in Central Chile, including Santiago, and the Mendoza and San Juan rivers, which are the main sources of water for the two largest cities (Mendoza and San Juan) in central-western Argentina. Seasonal variations in this region are shared by rivers on both sides of the Andes and normally show a minimum during the cold season followed by an early summer single discharge peak that largely reflects the winter snow accumulation levels (Fig. 2A). The second sub-region (PC2) explains 16.8% of the total variance and groups nine large river basins in the north-western Patagonian sector (Fig. 2B). The Andes in this sector are markedly lower and wetter than further north and most river discharges show a winter peak largely due to rainfall. However, in some cases a second spring peak can also be observed due to melt of the winter snowpack from the higher areas in these basins (Fig. 2B). This sub-region includes the large Bio Bio river in south central Chile, and the Neuquén and Limay rivers in Argentina, which collectively produce about 25% of the country’s hydropower generation^5^.

PC3 is formed by a group of five river catchments in the central-western Patagonian sector and explains 7.6% of the total variance in the dataset (Fig. 2C). The hydrological regime of the three rivers located further south in this sector (i.e. the Cisnes, Senguerr and Aisén basins; Fig. 2C) shows a single spring peak discharge due to snowmelt. In contrast, the two northernmost basins (Futaleufú and Palena) also display high runoff levels in winter. This is probably related to the relatively milder climatic conditions and the low elevation of the Andes in these northern basins, which receive most of the winter precipitation in liquid form. The fourth group identified by the PCA (PC4) is located immediately to the north of the first group, and explains 6.6% of the total variance. The group concentrates the four main river basins in the semi-arid Norte Chico region in Chile (Copiapó, Huasco, Elqui and Limarí; Fig. 2D) immediately to the south of the Atacama desert. Similar to their neighboring basins in PC1, the rivers in this fourth group have their greatest discharges in the early summer, concurrent with snowmelt from the high Andes. However, except for the Huasco basin, the rest of the rivers in the PC4 sub-region also experience a small cold season peak in discharge due to rainfall occurring at low elevations in the winter (see Fig. 2D).

**Figure 3:**
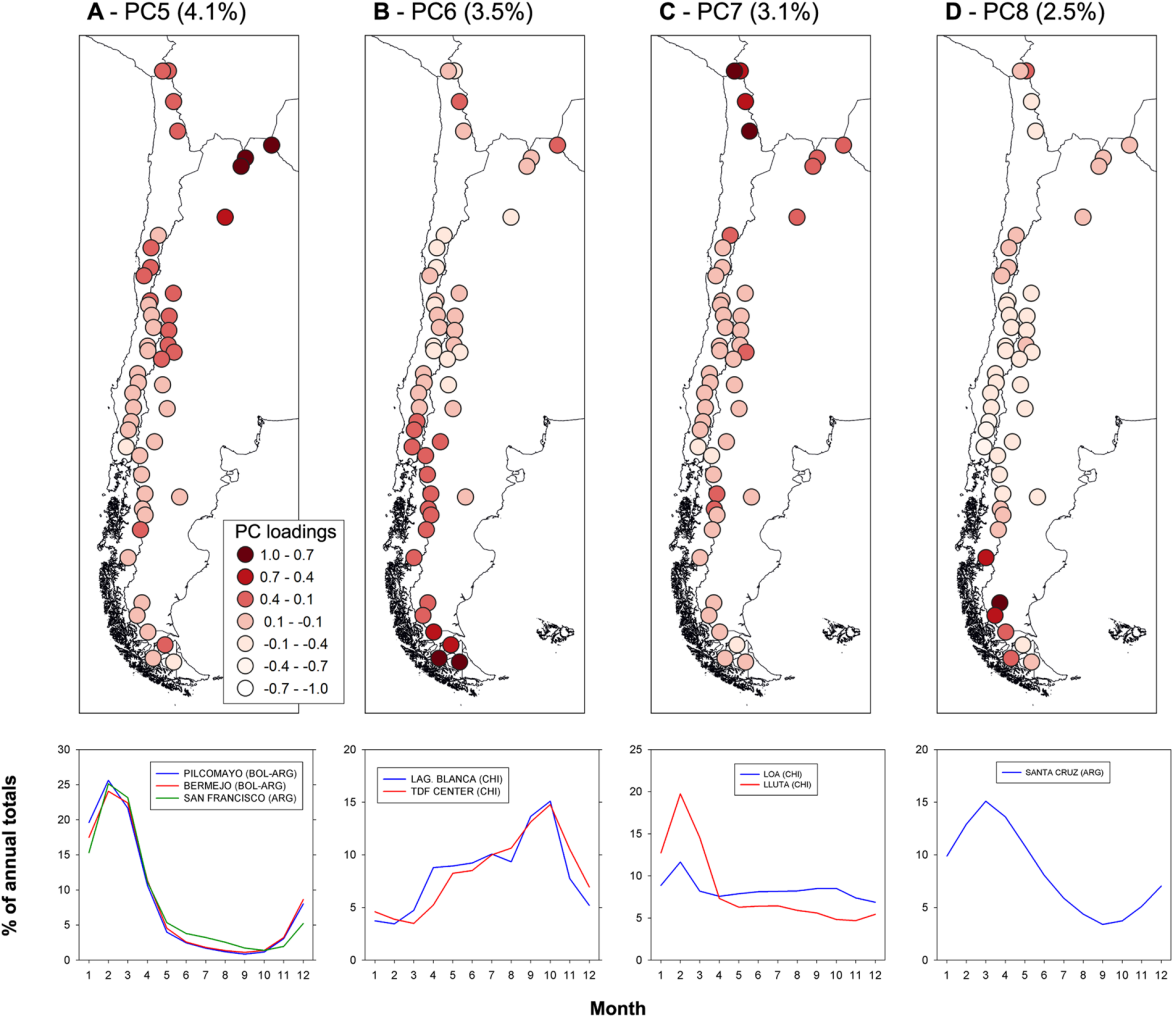
(**A** through **D**) Same as Fig. 2, but for PC5 through PC8, respectively.

The three most important rivers that drain the sub-tropical humid region of northwestern Argentina and southern Bolivia (the Bermejo, San Francisco and Pilcomayo rivers) were grouped into a fifth sub-region explaining 4.0% of the total variance in the dataset (PC5; Fig. 3A). The surface runoff in this region is largely concentrated in the austral warm season and reflects the concurrent annual peak in precipitation between December and February. Two rivers in the central Fueguian Andes, at the southern tip of the continent, form the sixth group identified by the PCA (PC6, with 3.4% of explained variance; Fig. 3B). In this case the rivers have a single, early spring peak in discharge largely due to snowmelt, but also high runoff values throughout the winter months. The seventh group detected by the PCA groups two river basins of the western Altiplano in northernmost Chile and explains 3.1% of the total variance in the dataset (PC7; Fig. 3C). In this extremely arid region surface runoff is quite limited, but the few small permanent rivers and creeks show a discernible peak discharge in summer that reflects occasional rainfall events during the warmer months.

The eighth group identified in the analyses (PC8) is formed by the individual mean monthly series of the Santa Cruz river (Fig. 3D). This component explains 2.5% of the total variance in the dataset. The Santa Cruz river drains the eastern portion of the South Patagonian Icefield and has a unique hydrological regime that is strongly influenced by the presence of the extensive glaciated areas located in the upper basin. Almost 3000 km^2^ (ca. 16%) of the Santa Cruz basin is covered by ice, representing a very rare and large proportion for an Andean catchment of this size. This particular configuration and the presence of two extensive proglacial lakes (the Viedma and Argentino lakes have a combined area of ca. 2500 km^2^) result in a very subdued seasonal regime with a single peak in the late summer – early fall largely dominated by glacier melt (Fig. 3D). Minimum discharges occur at the end of the winter before the onset of the melting season.

### Temporal variations in regional streamflow patterns

The analysis of the temporal variations of the regional time series corresponding to each principal component revealed contrasting modes of variability that reflect the enormous variety of hydro-climatic and environmental conditions that can be found in the southern Andes.

#### Mean monthly streamflow changes

**Figure 4:**
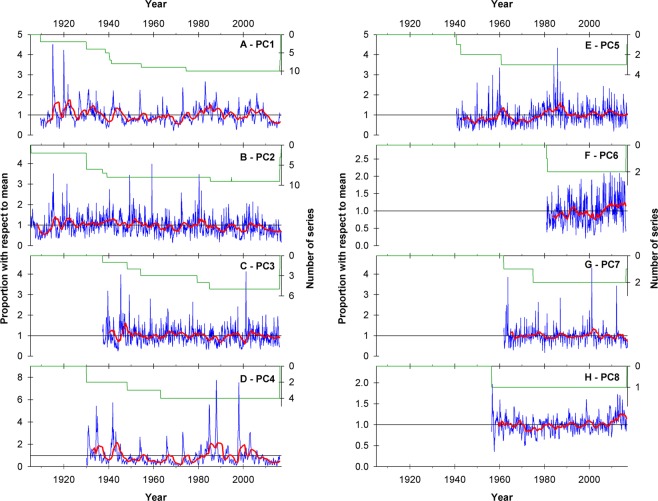
Regional mean monthly streamflow variations across the southern Andes. **(A)** Weighted monthly average of the station records with loadings >0.7 in PC1 (see Fig. 2). Running 36-month window means (red line) are depicted to emphasize the lower frequency patterns in the monthly series. The number of streamflow series used to calculate the weighted average is also shown (green line). Note that the scale in the right Y-axis is inverted. (**B** through **H)** Same as A, but for PC2 through PC8, respectively.

The PC1 record in the central Andes of Chile and Argentina covers the July 1909 – June 2016 period (1284 months with ≥ 2 series over the entire interval) and shows a strong intra- and inter-annual variability embedded in a marked multi-decadal pattern (Fig. 4A). This monthly record also shows several “spikes” that reflect sporadic increases in surface runoff which coincide with years of extreme high snow accumulation in the Andes. The neighboring, regionally-averaged record corresponding to PC2 in north-western Patagonia (Fig. 4B) is the longest available for the entire southern Andes and spans from May 1905 to December 2016 (1340 months with ≥2 series over the entire period). A notable characteristic of this time series is the existence of strong month-to-month variations (much larger, for example, than those observed in PC1) that reflect the wetter conditions and the higher importance of liquid precipitation modulating the surface runoff at these lower elevation basins.

The PC3 record centered in central-western Patagonia (Fig. 4C) is comparatively much shorter than the previous two series and spans over 949 months from April 1937 to April 2016 (≥2 series starting in April 1948). This record also aggregates fewer basins than the PC1 and PC2 regional series, but nonetheless it provides valuable instrumental hydro-climatic evidence for this poorly-known portion of the Patagonian Andes. Clear features of this record are the strong month-to-month variations (again reflecting much wetter conditions than, for example, in PC1) and a relatively neutral long-term trend over the entire period of instrumental measurements (Fig. 4C). The monthly time series corresponding to PC4 (Fig. 4D) is similar to that of PC1 but has a substantially larger range of variations around its long term mean. This series covers the interval January 1930 – April 2016 (>2 series starting in January 1930) and contains several spikes or sudden increases in surface runoff that only last a few months but can reach values over seven times above their corresponding monthly means. These extreme runoff events occur after several years with substantially lower flows, and are normally associated with extreme high snow accumulation in the Andes due to strong El Niño events in the tropical Pacific.

The monthly record that corresponds to PC5 in northwestern Argentina and southern Bolivia (spanning the September 1940 – August 2016 period, with >2 series since September 1942; Fig. 4E) shows a marked month-to-month variability which is characteristic of these humid basins. In contrast to the previous four regional series, the PC5 record shows a clear, overall positive trend over the period of record with several intervals portraying measurements above the long term mean. The PC6 mean monthly record from Tierra del Fuego also shows a positive trend in surface runoff during the period of available measurements (from December 1980 to April 2016, with two series from May 1981 onwards; Fig. 4F). This positive tendency in surface runoff is arguably the most notable feature of this short regionally-averaged record, where markedly higher values can be observed especially in the last 10–15 years of the series.

The mean monthly streamflow variations in the dry, western margin of the Altiplano in northernmost Chile are represented by the PC7 depicted in Fig. 4G. This series covers the interval November 1961 - December 2016, but until December 1974 it is formed by only one series. The runoff record from this arid region does not show a clear long-term trend, but has some months with very high values and a few extended periods that fluctuate above and below the long-term mean. Finally, the monthly record corresponding to PC8 spans over the June 1956 – August 2016 period (Fig. 4H) and is formed by only one series, i.e. the monthly record from the Santa Cruz river alone. This series shows a highly variable pattern with a negative tendency during the first two decades of record, followed by an overall increasing trend from the early 1970s until recent times.

#### Annual variations and extreme period analyses

**Figure 5:**
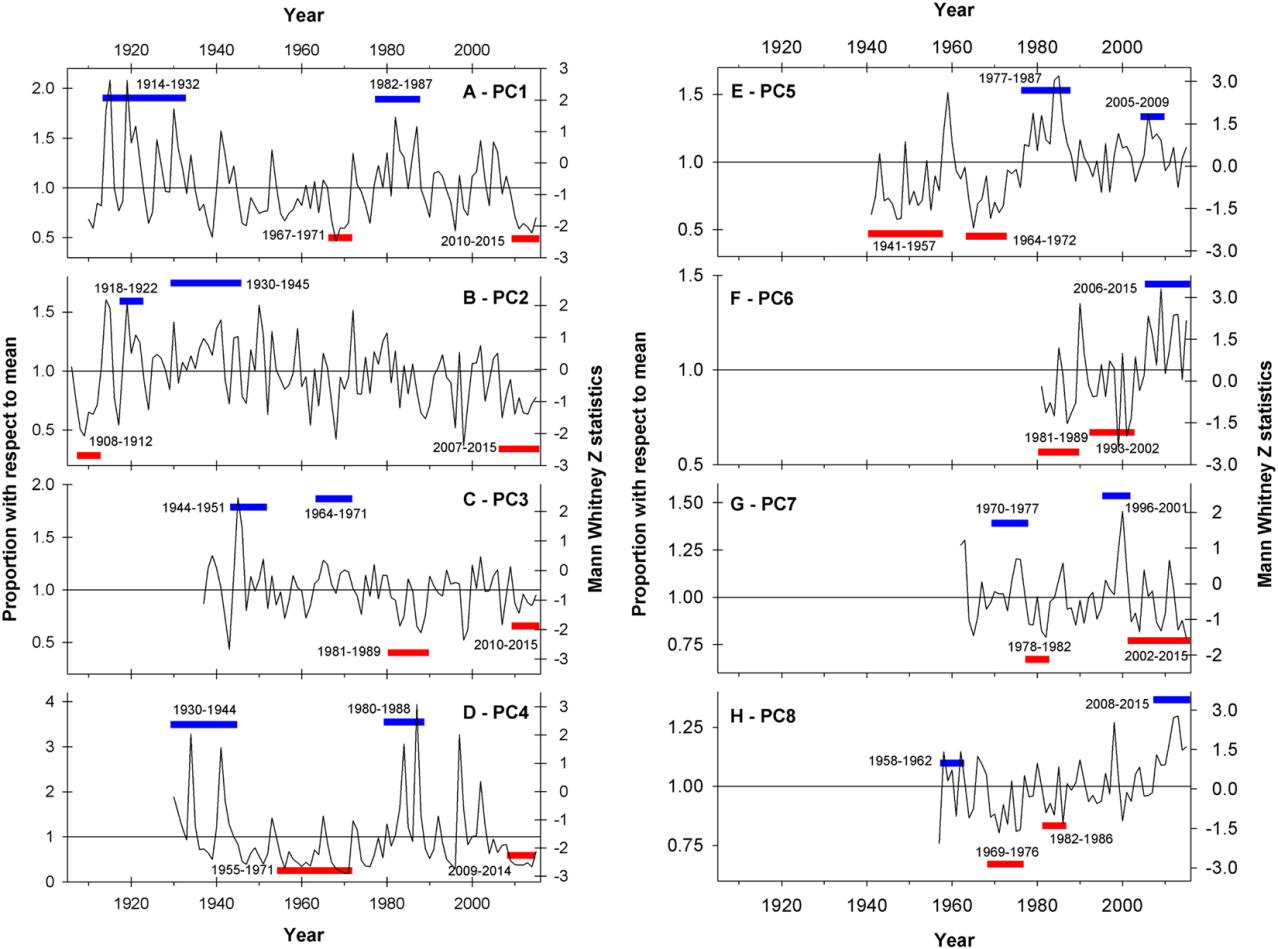
Regional mean annual changes in Andean surface runoff. **(A)** Mean annual (April-March) variations of the PC1 region calculated using the monthly series shown in Fig. 4. Periods with the highest (lowest) MWZ values of all possible moving windows of 5–20 years in length are indicated by the blue (red) horizontal bars (see Data and Methods for details). (**B** through **H)** Same as A, but for PC2 through PC8, respectively.

The annualized (April-March) time series corresponding to PC1 shows an overall negative trend since the early 20^th^ century (Fig. 5A). Within this negative trend, the analyses of the wettest and driest periods among all non-overlapping 5–20 year windows indicates that they occurred during 1914–1932 and 2010–2015, respectively. Extreme wet (dry) conditions were also identified in 1978–1987 (1967–1971; Fig. 5A). All these extreme periods identified by the non-parametric Mann-Whitney/Monte Carlo analyses are statistically significantly different (at the 95% confidence level) when compared to an essentially stationary time series with the same variance and autocorrelation of the observed record (see Data and Methods section for details). As observed in the PC1 time series, the PC2 record also shows a marked (and even clearer) negative long-term trend that is particularly noticeable at these annual timescales (Fig. 5B). The driest period of the series occurred in 1908–1912 and is statistically significant at the 99% level. The second driest period (95% significance) took place in the last nine years of record between 2007 and 2015. In contrast, the wettest intervals occurred in 1930–1945 and 1918–1922, and are statistically significant at the 99% and 95% confidence levels, respectively (Fig. 5B).

The annualized record corresponding to PC3 fluctuates around the long term mean without a clear tendency towards drier or wetter conditions between 1937 and 2015 (Fig. 5C). Within this neutral trend there are, however, several periods that stand out as significantly drier (or wetter) than expected in stationary conditions. In this Patagonian region the two wettest periods (significant at the 95% level) occurred in 1944–51 and 1964–1971, whereas the driest intervals took place during 1981–1989 and 2010–2015 (99 and 90% significance level, respectively; Fig. 5C). The annualized record from the PC4 region (Fig. 5D) displays a clear multi-decadal pattern and a substantially greater range of year-to-year variations than the previous regional series. In the case of this PC4 record, the wettest intervals occurred in the 1930–1944 and 1980–1988 periods (95% significance in both cases), whereas extended dry conditions took place during 1955–1971 and 2009–2014 (99% and 95% significance, respectively).

The annualized series corresponding to PC5 (Fig. 5E) is characterized by a strong positive trend, with a much greater frequency of below (above) average flows in the first (second) half of the record, and a transition marked around the late 1970s. The analyses of the most extreme periods of this record support this positive trend and show that the lowest flows occurred during the first half of the record (i.e. 1941–1957 and 1964–1972, with 95% statistical significance), and the highest flows in 1977–1987 and 2005 and 2009 (99% and 90% significance, respectively; Fig. 5E). A strong positive trend is also observed in the short annual record from Tierra del Fuego (PC6; Fig. 5F). In this case the 2006–2015 interval was identified as the wettest on record (99.9% significance), whereas the 1981–1989 period showed the lowest flows (95% significance). The analyses also identified the 1993–2002 interval as the second driest on record with a 90% statistical significance (Fig. 5F).

The annual PC7 record shows practically no trend over the 1962–2015 period (Fig. 5G). Within this trendless pattern, however, the intervals 1970–1977 and 1996–2001 were identified as the wettest on record, with a 90% and 95% significance, respectively. In contrast, the driest periods on record occurred in 1978–1982 and 2002–2015 and reached a 95% and 90% statistical significance (Fig. 5G). Moving south, the annualized series from the Santa Cruz river in southern Patagonia (PC8; Fig. 5H) has experienced a marked increase in annual flows during the last decades. This is particularly evident in the extreme period analyses, which showed that the wettest (driest) interval occurred between 2008 and 2015 (1969 and 1976) and reached 99.9% (99%) statistical significance. These tests also revealed that 1982–1986 was the second driest interval but not statistically significant even at the 90% confidence level. Similarly, the second wettest interval of this record (1958–1962) was not statistically significant either (Fig. 5H).

#### Multi-decadal modes of variability

**Figure 6:**
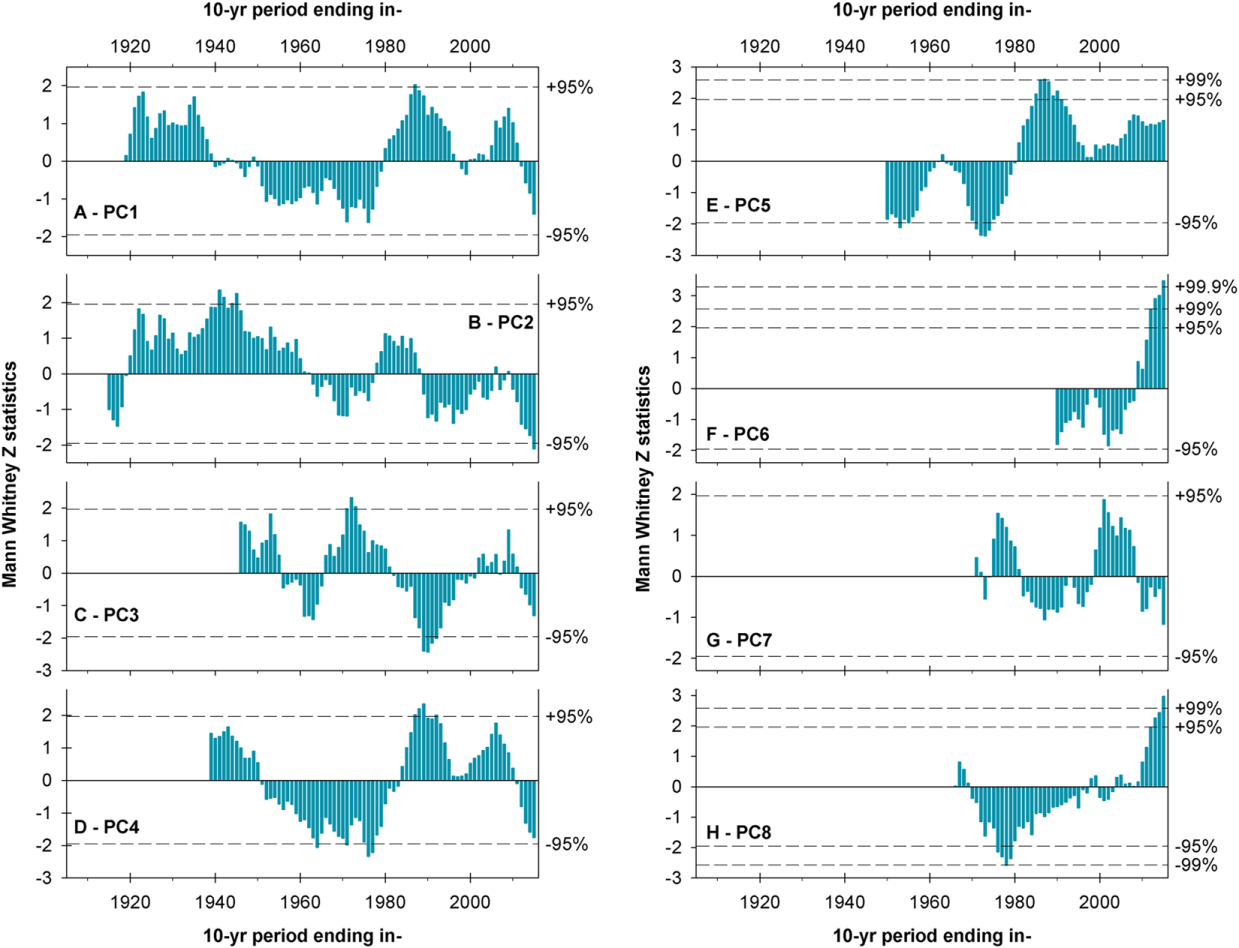
Multi-decadal patterns of streamflow variations in the southern Andes. **(A)** Mann-Whitney Z statistics for running 10-year samples of the mean annual, regionally averaged PC1 record shown in Fig. 4. Horizontal dashed lines indicate positive and negative significance at different confidence levels (see Data and Methods for details). (**B** through **H)** Same as A, but for PC2 through PC8, respectively.

When aggregated into decadal values, the regional records described above exhibit marked, distinctive low frequency patterns of hydro-climatic variability for the different sectors of the southern Andes. The decadal moving averages corresponding to PC1 (Fig. 6A) show above-average conditions in the 1920s and 1930s, an extended dry period between the 1940s and the 1970s, and substantially higher runoff values again in the 1980s and part of the 1990s. During the 21^st^ century this record showed above average flows until 2010, and since then a marked decline that has continued until recent times. Examined at this decadal timescale, however, only one decade in the late 1980s reach the 95% significance level (Fig. 6A). In contrast, the decadal PC2 series shows several extreme wet, statistically significant decades in the late 1930s – early 1940s followed by an unequivocal negative trend that culminates with the driest decade on record between 2006 and 2015 (significant at the 95% confidence level; Fig. 6B). The decadal series from the PC3 region (Fig. 6C) shows a quasi-cyclic pattern alternating between wet and dry intervals of 10–20 years in length, and culminates in negative (yet non-significant) values. However, note that when this series was examined using the extreme period routine (see Fig. 5C above), these most recent years were classified among the driest on record.

The low frequency patterns in the PC4 decadal series (Fig. 6D) are similar to those observed in PC1, but the highs and lows in the PC4 series are more extreme and the overall negative trend is more clearly discernible than in PC1 (see Fig. 6A). The pattern observed in the decadal PC5 series, on the contrary, shows a marked positive trend and several decades between the 1950s and 1970s that are statistically significantly different (lower) than expected by chance alone (Fig. 6E). This figure also shows that since the late 1970s – early 1980s all decadal values have remained above the long-term mean. The values recorded in the 1980s represent the highest flows on this record, with some of them reaching the 99% significance level.

The analyses of the PC6 decadal record show that the last 10 years (2006–2015) were remarkably wet, with values 99.9% significantly higher than the “expected” flows (Fig. 6F). Unfortunately, the reduced number of stations and the shortness of the available streamflow series in this southernmost region preclude the analysis of earlier patterns (i.e. prior to 1981), and thus it is unclear if these unusually high recent streamflow levels have occurred in the past in this region. Moving to northernmost Chile, the decadal time series from the PC7 region (Fig. 6G) shows that none of these moving windows are significantly different than expected by chance alone at the 95% confidence level. In contrast, the decadal time series of PC8 shows several moving decades outside the significant thresholds and is dominated by an increasing trend that began in the 1970s and continued unabated until 2015 (Fig. 6H). In statistical terms, four moving averages in the late 1970s have values that are lower than expected at 95% statistical significance. This is also true for the last 3–4 values of this record, where the last decade (2006–2015) can even be considered significantly different (higher) than expected with 99% statistical significance (Fig. 6H).

### Relationship with large-scale ocean-atmospheric indices

The multivariate regression trials revealed that the indices reflecting the variations of the ENSO phenomenon (i.e. N34, SOI and MEI, see Supplementary Table 2) are those more strongly associated with the annual river flows in the PC1, PC2, PC3 and PC4 regions (Table 1). In the case of PC1, the PDO and the AMO also show significant correlations with streamflows, but due to the intercorrelation among the indices only the MEI was included in a regression model which explains about 41% of the runoff variance (Table 1). The same indices are also statistically significantly correlated with the PC2 series, but in this case the list also includes the AAO with a negative correlation coefficient of −0.545 (significant at the 99% confidence level). Nonetheless, in this case only the SOI record passed the stepwise regression procedure and was included in a model that explains ca. 45% of the variance in this regional streamflow record (Table 1). The model developed for the PC3 record explains ca. 36% of this Patagonian pattern and includes an ENSO-related index (N34; Table 1) but also the SAM as significant predictors. In the case of PC4, the percentage of variance explained by the regression model decreases to ca. 20% and only includes the MEI as significant predictor.

**Table 1:** Multivariate regression trials between large-scale climatic indices and the regional streamflow records (PC1 to PC8).

Climate index	PC1			PC2			PC3			PC4		
	*r*	*Beta*	*Adj R*^2^	*r*	*Beta*	*Adj R*^2^	*r*	*Beta*	*Adj R*^2^	*r*	*Beta*	*Adj R*^2^
N34	0.605**	−0.581	0.410	0.646**	0.055	0.446	0.527**	0.473**	0.361	0.403**	−1.086	0.201
SOI	−0.593**	0.061		−0.680**	−0.680**		−0.424**	0.585		−0.346*	0.608	
MEI	0.654**	0.654**		0.613**	−0.091		0.447**	−0.997		0.474**	0.474**	
PDO	0.323*	0.000		0.297*	0.097		0.139	−0.056		0.427**	0.255	
AMO	−0.293*	0.088		−0.545**	−0.205		−0.521**	−0.134		−0.072	0.263	
TSA	−0.105	−0.003		−0.121	0.002		−0.220	−0.041		0.169	0.248	
SAODI	−0.274	−0.079		−0.130	0.024		−0.244	−0.034		−0.027	0.133	
AAO	−0.225	−0.089		−0.302*	−0.155		−0.421**	0.190		−0.175	−0.077	
SAM	−0.161	−0.091		−0.262	−0.174		−0.425**	−0.353*		−0.117	−0.066	
	**PC5**			**PC6**			**PC7**			**PC8**		
	***r***	***Beta***	***Adj R***^**2**^	***r***	***Beta***	***Adj R***^**2**^	***r***	***Beta***	***Adj R***^**2**^	***r***	***Beta***	***Adj R***^**2**^
N34	−0.093	0.072	0.128	0.202	0.150	0.105	−0.325*		#	−0.264	−0.181	0.127
SOI	0.091	−0.033		−0.116	−0.068		0.327*			0.292*	0.214	
MEI	−0.171	0.031		0.224	0.187		−0.291*			−0.249	−0.173	
PDO	−0.393*	−0.393*		0.165	0.165		0.001			−0.344*	−0.295	
AMO	−0.115	−0.203		−0.146	0.039		0.062			0.332*	0.188	
TSA	0.092	0.124		−0.083	0.048		−0.327*			0.195	0.045	
SAODI	0.282	0.191		−0.222	−0.122		−0.283*			0.127	−0.021	
AAO	−0.060	−0.116		−0.341*	−0.009		−0.156			0.392*	0.392*	
SAM	−0.035	−0.035		−0.363*	−0.363*		−0.115			0.365*	−0.014	

The tests were performed on the first-differenced, April-March annual averages over the 1982–2015 period common to all series. Candidate predictors among the climatic indices were selected following a stepwise regression approach (F-to-enter 0.05, F-to-remove 0.10). Notes: (N34) Niño 3.4 index; (SOI) Southern Oscillation Index; (MEI) Multivariate ENSO Index; (PDO) Pacific Decadal Oscillation; (AMO) Atlantic Multidecadal Oscillation; (TSA) Tropical South Atlantic Index; (SAODI) South Atlantic Ocean Dipole Index; (AAO) Antarctic Oscillation; and (SAM) Southern Annular Mode (see Supplementary Table 2 for further details). (*r*) Pearson correlation coefficient between the streamflow series and each climatic index; (*Beta*) standardized regression coefficients used to compare the relative strength of the various predictors within each model; (*Adj R*^2^) coefficient of determination adjusted for the number of predictors in the model. The (*) and (**) symbols denote statistical significance at the 95% and 99% confidence levels, respectively, and the (#) symbol indicates that none of the predictors passed the threshold value for F-to-enter.

The regression models developed for the remaining regional series (i.e. PC5 to PC8) show a markedly lower skill in predicting annual flows than those obtained for the previous PCs (Table 1). The PC5 (PC6) record is only statistically correlated at the 95% with the PDO (the Antarctic indices AAO and SAM), and due to the relatively weak association between these variables, only ca. 13% (11%) of the streamflow variance is accounted for by the resulting model. Interestingly, although the indices from the tropical Pacific (N34, SOI and MEI) and the south Atlantic Oceans (TSA and SAODI) show significant associations with the PC7 record, none of these predictors is strong enough to pass the threshold value for F-to-enter and thus no regression model was constructed for this regional series (Table 1). Finally, the correlations tests with the PC8 record in southern Patagonia show several significant associations with indices from the Pacific, Atlantic, and Southern Oceans. However, only the AAO was included in a regression model that explains a marginal portion (ca. 13%) of the total variance in this record (Table 1).

## Discussion and Conclusions

The compilation and aggregation of basic instrumental hydrological data from the Chilean, Bolivian and Argentinean Andes, and their subsequent statistical processing and analysis, revealed the existence of valuable (and often overlooked) station records that can provide reliable hydro-climatic information for practically the entire Andean region south of 18°S. Some of these records have been discontinued but proved very useful for extending the interval under study, and also for estimating monthly variations during periods when some reference series showed gaps in available information. Nonetheless, the large number of available records (1226 in total) and their great heterogeneity in length and temporal coverage required several measures to obtain a reasonable number of long and representative time series that could be used to characterize the main hydro-climatic patterns across the region. For this, all suitable records (i.e. those with ≥60 months and minimal anthropogenic influence, totaling 513 station records) were first assessed statistically and then aggregated to reduce the number of series under investigation. This procedure resulted in single, serially complete monthly runoff records for 51 major river basins in the Andes between 18° and 55°S. The number of series under study was further reduced by the application of a Principal Components Analysis routine, which identified only eight distinctive modes of variability and allowed a better assessment and intercomparison of the main streamflow patterns at local and regional scales. This statistical processing and the resulting time series offer important new insights that are particularly relevant considering the scarcity and shortness of high elevation meteorological data in this mountain chain, which encompasses a latitudinal range of >4000 km with extreme contrasts in climate and environmental conditions. The strong common signal found in most of the instrumental records from within each major river basin (and also from adjacent basins) was another interesting finding. It ultimately reflects the overall good quality of this rich but largely underutilized hydrological dataset, which has been maintained for decades by numerous individual operators and official institutions in Chile, Argentina and Bolivia.

The use of runoff records that are relatively up-to-date, combined with time series analysis routines specifically designed to detect the location, extension, and statistical significance of the most important low-frequency patterns in the streamflow records also allowed the assessment of the most recent period in a long-term, multi-decadal context. In this case the procedure included the identification of the highest and lowest ranked annual mean values (i.e. those more statistically significant) for all possible moving windows of 5–20 yrs in length in each regional series, and the evaluation of the statistical significance of all 10-yr moving windows in the eight regions identified by the PCA. These tests showed, for example, that during the last several years the river discharges in the regions identified by PC1, PC2, PC3, PC4, and PC7 have been statistically significantly lower than expected by chance alone (see Fig. 4A–D,G, respectively). In contrast, the sectors identified in PC5, PC6, and PC8 have all experienced during approximately the same interval surface runoff values that are significantly higher than normal in statistical terms (see Fig. 4E,F,H, respectively).

These findings and the other high- and low-frequency patterns of hydrological variability discussed above provide a first general approximation to a comprehensive understanding of the complex hydro-climatic system of the southern Andes. However, it should be noted that, for some sectors of the study area, and particularly those with semiarid climates where the Andean water resources are crucial for the subsistence and development of human activities, the recent runoff variations are relatively well known as the issue of reduced water availability and the need for better water management strategies have been addressed by a growing number of studies in recent years (see e.g. refs. ^6–8^). Probably the clearest example is the extreme dry period that started in 2010 (locally known as the “megadrought”^8^), which is being monitored with concern on both sides of the Andes as it continues unabated in the PC1 and part of the PC2 and PC4 regions. Our results agree with those from recent assessments^7,8^ placing the period after 2009–2010 in these regions among the driest on record, with earlier droughts of similar magnitude only occurring more than 40 years ago (see Figs. 5 and 6). Interestingly, when assessed in a much longer, multi-century context derived from a tree-ring based precipitation reconstruction^8^, this extended dry period appears to have very few analogs during the last millennium.

Recent efforts have also attempted to analyze the streamflow patterns for the entire Andes cordillera^9,10^ but these large-scale assessments have only tackled the Pacific (western) draining basins in the Andes and are mainly based on a modeling approach. These results depend on several assumptions and are constrained by the inherent uncertainties of the models and their input data^10^. In this sense, the statistical analyses and results presented here, which are entirely based on direct *in-situ* streamflow measurements, can serve as a very useful baseline and validation dataset from which other assessments of increasing complexity can be developed (see e.g. ref. ^1^). Our results can also be used to better define the spatial extent of the main modes of hydrological variability and the boundaries between Andean regions with different hydrological regimes. In south central Chile (34°–45°S), for example, the boundary between PC1 and PC2 identified here largely agrees with a previous regionalization of streamflow records based on a clustering procedure^11^. However, the limited spatial extent of most previous studies precludes a proper identification and definition of the different hydrological patterns, and to our knowledge the present paper is the first study that provides such evidence for the entire Andean region south of 18°S.

Several other studies have assessed the relationship between Andean river flows and large-scale ocean-atmospheric indices such as ENSO or the PDO (see e.g. refs. ^11–13^, and references therein). These assessments provide important information for an improved understanding of the influence of these indices on Andean hydrology, but in general their results are focused on specific sectors of the southern Andes. The analyses discussed in the present manuscript complement these previous efforts providing a comprehensive overview that includes the entire Andean region south of 18°S, facilitating the inter-comparison between regions. In this context, the ENSO phenomenon appears as an important forcing modulating annual streamflow variations in Andean catchments between ca. 28° and 46°S (more specifically, in the PC1, PC2, PC3 and PC4 regions; see refs. ^11,12^ and Table 1). The PDO, widely used to reflect the multi-decadal variability in the Pacific Ocean, is statistically associated with flow variations in the PC1, PC2, PC3 and PC5 regions, but only in the latter it can be considered a significant (albeit weak) predictor of annual flows (Table 1). In contrast, and despite showing statistically significant correlations with various regional streamflow patterns in the Andes, none of the indices associated with large-scale variations in the Atlantic Ocean (i.e. AMO, TSA or SAODI, Table 1) was selected as a significant predictor of annual flows in the stepwise regression procedure. The multivariate regression results also show that the indices portraying ocean-atmospheric variability around Antarctica (AAO and SAM) are statistically associated with Andean flows. However, this association is mostly concentrated along the Patagonian and Fueguian Andes (i.e. in the PC2, PC3, PC8 and PC6 regions), and their predictive skill of annual flows is comparatively weak: Only in the two southernmost regions (PC8 and PC6) can the Antarctic indices be considered significant predictors of annual flows, and even in these cases the percentage of explained variance accounted for by the models ranges around 11–15% (Table 1).

More detailed studies are required to increase understanding of the forcings behind the streamflow patterns discussed above. These detailed assessments are outside the main goals of the present study, but could include more specific statistical tests and modeling approaches to differentiate the relative influence of these large-scale atmospheric indices at seasonal and monthly timescales. The lagged correlation of these indices with Andean flows should also be considered, especially in those basins where most of the discharge results from the melting of snow accumulated in previous months. The organization and formatting of the surface runoff records discussed here (i.e., serially complete and up-to-date mean monthly time series aggregated for most of the major hydrological catchments in the southern Andes) should facilitate these large-scale assessments.

The format and regional representativeness of these records should also be of relevance for related studies dealing with the reconstruction of surface runoff variations using high-resolution proxy records such as tree rings or varved lake sediments. Some promising results based on tree-ring records have been already explored in the literature (see e.g. refs. ^14–17^), and the time series presented here could complement and expand geographically the potential for future studies in the southern Andes. Lastly, another potential application of the records developed and discussed in this study includes the comparison with other independent indicators of hydrological conditions across large areas. In this sense the information on changes in terrestrial water storage observed by the Gravity Recovery and Climate Experiment (GRACE) satellites^18^ appears to be a very interesting alternative for comparing and validating the hydrological variations determined by direct measurements in the Andean regions. Not only the large size of the units sampled by the GRACE instruments (hundreds of kilometers) is roughly in line with the size of the hydrologic regions identified by the PCA approach, but also the temporal resolution of both datasets is similar for a proper and relatively straightforward intercomparison of these independent records. Although the potential sources of error and the non-climatic influencing variables should be considered carefully and removed from the GRACE series prior to analysis^18,19^, the new streamflow dataset and the regional aggregation presented in this study could provide valuable information for this and many other related assessments, hopefully contributing to a much better understanding of the main processes regulating the mountain hydrology in the Andes.

## Data and Methods

### Initial filtering of mean monthly streamflow records

Mean monthly streamflow records from Andean basins south of 18°S were assembled from official databases from Chile, western Argentina and southern Bolivia (see Fig. 1B and Supplementary Table 1 for details). Prior to analysis, stations with series shorter than 60 months were removed together with those located downstream from dams, heavily populated areas, irrigated farmlands and/or industrial centers that could have affected the natural streamflow variations measured at the gauging station. This filtering procedure excluded 713 stations (527 from Chile, 174 from Argentina, and 12 from Bolivia) and was particularly important in Chile because of the high density of gauging stations in this country; it effectively removed many records from lower elevations (see Fig. 1B,C). The final dataset consists of 513 stations which have the longest series with relatively natural regimes south of 18°S in the Andes (282 from Chile, 229 from Argentina, and 2 from Bolivia; Fig. 1C and Supplementary Table 1). Collectively, the contributing areas located upstream from these 513 stations cover almost the entire Andean region from 18° to 55°S. Within this region practically all major river basins are represented by at least one streamflow series. It is important to note that given the large number of records and environmental conditions that are considered in this selected dataset, it is practically impossible to isolate and remove all the possibly remaining anthropogenic influences from these records. Nonetheless, it should be noted that the analyses below are largely focused on regionally averaged series, which enhance the common signal contained in the series and reduce the impacts of any potential inhomogeneity in the individual station records.

### Development of representative streamflow series for individual river basins

The selected monthly records were first deseasonalized (by dividing the individual mean monthly values by their long term monthly means) to remove the cyclic nature of the data, and then log-transformed to minimize the positive skewness that is common in hydrological records^20^. Subsequently, the longest and most complete records (>20 yrs of data, reaching at least to the year 2010) from each major river system in the study area were selected to develop one reference mean monthly streamflow series per basin. If necessary, the missing months in these reference series were infilled with a weighted average of values from well-correlated neighboring stations within the same basin (see percentage of missing data that were infilled with this procedure in Supplementary Table 1). The weighted averages were computed using series that correlate at the 99.9% significance level and share at least 60 months with the reference series. Periods ≤18 months where no monthly values were available from either the reference series or the weighted average of neighboring stations were infilled with mean values (i.e. with zeroes in the deseasonalized and log-transformed series). If the data gaps were >18 months, the early portions of the records were discarded from the analyses. This procedure resulted in serially complete mean monthly streamflow records for 51 major river basins or sectors across the southern Andes (Fig. 1D and Supplementary Table 1).

### Identification of the main spatial patterns in monthly streamflow records

The main patterns of spatial variability in surface runoff in the southern Andes were identified using obliquely rotated Principal Component Analysis^21^ (hereafter PCA). Extensive testing of several principal component rotation methods^22,23^ revealed that obliquely rotated solutions perform significantly better than unrotated and orthogonally rotated methods in objectively decomposing spatial arrays of real and simulated climate data into regional clusters or patterns. Since obliquely rotated solutions do not constrain orthogonality of the vectors, they tend to reflect the natural regional patterns of common variability present in the original records in a more physically realistic manner^21,23^. Following the recommendation of [^22^] we used an oblique Promax rotation^24^ with a power k = 2. The analyses were performed using a correlation input matrix in which all series are equally weighted. Here the resulting map types are not biased by areas of high and low variance and therefore will not concentrate the synoptic centers in areas of maximum variance^21^. This may be of relevance given the large size of the study area and the inherent variability observed in the records, where for example some stations in central-northern Chile show three to four times larger variability than records located further south in the Andes. Significant components were identified as those with eigenvalues ≥1 (ref. ^25^). Given the uneven temporal coverage of the records (Supplementary Table 1), for the identification of regions with common hydroclimatic variability we used the April 1986 to March 2016 period (360 months) which is shared by 48 of the 51 surface runoff series in the final dataset.

### Creation of regionally averaged streamflow series

The results from the PCA were subsequently used as the basis to develop regionally averaged mean monthly streamflow series that characterize the main temporal modes of hydroclimatic variability in the southern Andes. These regionally representative time series were created using a weighted average (weighted by their component loadings) of the stations with component loadings > 0.70 in each principal component identified in the surface runoff data. As the principal component loadings can be interpreted as simple correlation coefficients between each factor and the original variables, this threshold value ensured a strong common signal for each regional record and avoided the overlap between regions. Compared to using the common time interval selected in the PCA (i.e. April 1986 - March 2016), this averaging approach also has the advantage of maximizing the information available from each region and allows a further extension of the records by combining highly correlated series covering different time intervals. After averaging, the regional monthly series were back transformed and expressed as proportions from their long term mean.

### Identification of the main temporal modes of streamflow variability

To characterize the most important low frequency temporal patterns and evaluate the severity of the most extreme periods in each region, we first annualized the regionally averaged mean monthly values using an April-March hydrological year, and then applied a nonparametric time series analysis procedure that uses a combination of Mann-Whitney U tests^26,27^ and Monte Carlo simulations to provide significance levels for moving time windows of varying duration in hydroclimatic series^28–30^. Essentially this method samples data rankings over moving time windows (ranging here between five and 20 yrs in length), converts those samples to Mann Whitney U statistics, and then normalizes the U statistics to Z statistics using Monte Carlo generated null parameters. Based on the magnitude of the Mann–Whitney Z values this algorithm can identify time windows containing significant incidences of low or high data rankings, where Z values of ±1.960, ±2.576 and ±3.291 represent the 2-sided 95%, 99% and 99.9% confidence levels, respectively. This technique offers an objective, relatively simple, and statistically based way of detecting the driest and wettest intervals in hydrological time series from a given region^28–30^.

### Testing the relationships with large scale ocean-atmospheric indices

We also performed exploratory multiple regression analyses using several ocean-atmospheric indices as potential predictors of the streamflow patterns discussed above. These indices (see Supplementary Table 2) are commonly recognized among the main forcings modulating hydro-climatic conditions across southern South America, and were thus considered possible predictors of inter-annual variations of surface runoff in the southern Andes. Prior to regression, all monthly time series were converted to annual averages using the April-March hydrological year. Then they were converted to first-differences (subtracting to the annual values of year *t* those from *t* – 1) to minimize the spurious influence that long-term trends and serial correlation may have of the regression results^31^. As the variations of most of these large-scale indices are not statistically independent, a stepwise regression approach (F-to-enter 0.05, F-to-remove 0.10) was used to avoid multicollinearity among the predictors. This approach allowed isolating the partial influences of each candidate predictor on inter-annual streamflow variations during the 1982–2015 interval common to all series.

## Supplementary information

Supplementary Material

## Data Availability

The original mean monthly streamflow records and the climatic indices are freely available from the official sources (see Supplementary Tables 1 and 2 for details). The serially-complete mean monthly streamflow series for the 51 reference basins, and the resulting regional composites (PC1 to PC8), are available upon request from the first author.
