# Supplementary material for: Streamflow variations across the Andes (18°–55°S) during the instrumental era
Source: Sci Rep. 2019 Nov 29;9:17879. doi: 10.1038/s41598-019-53981-x (PMC6884640; doi:10.1038/s41598-019-53981-x)
Supplement: Supplementary file 1 — Supplementary Material [file 41598_2019_53981_MOESM1_ESM.pdf]

Supplementary Material for the paper "Streamflow variations across the Andes (18°-55°S) during the instrumental era"

M.H. Masiokas[1*]; L. Cara[1]; R. Villalba[1]; P. Pitte[1]; B.H. Luckman[2]; E. Toum[1]; D.A. Christie[3,4]; C. Le Quesne[3]; S. Mauget[5]

[1] Instituto Argentino de Nivología, Glaciología y Ciencias Ambientales (IANIGLA),

CCT- CONICET Mendoza, C.C. 330, (5500) Mendoza, Argentina.

[2] Department of Geography, University of Western Ontario, Canada.

[3] Laboratorio de Dendrocronología y Cambio Global, Instituto de Conservación Biodiversidad y Territorio, Facultad de Ciencias Forestales y Recursos Naturales, Universidad Austral de Chile.

[4] Center for Climate and Resiliance Research (CR)^2, Chile.

[5] Wind Erosion and Water Conservation Unit, Agricultural Research Service, U.S. Department of Agriculture, Lubbock, Texas, USA.

* Corresponding author email address: mmasiokas@mendoza-conicet.gob.ar

Tel: +54-261-5244264

Fax: +54-261-5244001

Supplementary Table 1. Selected stations used in this study, ordered from north to south (see Fig. 1C).

Sources of data:
(1) Dirección General de Aguas, Chile (http://www.dga.cl).
(2) Secretaría de Infraestructura y Política Hídrica, Argentina (https://www.argentina.gob.ar/interior/secretaria-de-infraestructura-y-politica-hidrica/base-de-datos-hidrologica-integrada).
(3) Comisión Trinacional para el Desarrollo de la Cuenca del Río Pilcomayo (Bolivia, Paraguay and Argentina; https://www.pilcomayo.net/).
(4) Pers. comm., Dr. Zulma Menna and Omar Castillo (Universidad Nacional de San Juan, Argentina).

Notes:
(*) Stations highlighted in yellow were selected as reference sites to represent their basins in subsequent analyses, with the numbers corresponding to the order indicated in Fig. 1D.
(**) The numbers in parentheses represent the Principal Component assigned to each station after assessing these reference sites over the April 1986 – March 2016 period (see Data and Methods section for details).
(#) The available Bolivian records consist of mean daily water levels and sporadic direct discharge measurements for gauging stations from the Pilcomayo river basin. For each of these stations we first calculated rating curves that relate specific water heights to mean daily discharges, and then aggregated the daily discharge values into mean monthly streamflow records.
(†) The Caimancito station at the outlet of the San Francisco river basin in northwestern Argentina is located downstream from the city of San Salvador de Jujuy. This station contains the longest, most complete and up-to-date river discharge record in this extensive and data-poor basin. Although the water demands for the city and the adjacent cultivated fields may have influenced the natural regime of Río Grande (one of the tributaries of the San Francisco river), the comparison of the monthly Caimancito time series with records from neighboring basins suggests that the overall anthropogenic impact is minimal at this station.
(‡) In the Limay and Senguerr river basins in southern Argentina, the weighted averages were constructed using not only records from within these basins, but also from the three best correlated records from adjacent basins. For the Limay record this allowed extending its long 1903-1990 series up to the present. In the case of the Senguerr river, the inclusion of neighboring records allowed connecting the most recent period of measurements (1984-2016) with earlier records collected at the same site between 1949 and 1976. Without this approach the valuable earlier records from this poorly known Patagonian region would have been excluded from analysis.

ID	Country (Source)	Station	Basin	Lat.	Long.	Elev.	Interval	N	% Miss.	Ref. * (PC)**
1201005	Chi (1)	Río Caracarani En Humapalca	Lluta	-17.85	-69.70	3908	1973-8 / 2017-6	510	6.1	
1201002	Chi (1)	Río Caracarani En Alcerreca	Lluta	-17.98	-69.63	3253	1961-12 / 1997-9	121	72.7	
1201001	Chi (1)	Río Colpitas En Alcerreca	Lluta	-18.00	-69.63	3251	1961-12 / 2016-10	488	28.1	
1001002	Chi (1)	Río Caquena En Vertedero	Altiplanicas	-18.00	-69.26	4272	1969-12 / 2016-10	461	20.5	1 (None)
1201003	Chi (1)	Río Lluta En Alcerreca	Lluta	-18.00	-69.63	3550	1961-11 / 2017-6	552	19.8	2 (7)
1001003	Chi (1)	Río Colpacagua En Desembocadura	Altiplanicas	-18.02	-69.24	4302	1988-6 / 2016-9	291	16.9	
1001001	Chi (1)	Río Caquena En Nacimiento	Altiplanicas	-18.08	-69.20	4385	1976-7 / 2004-5	247	28.6	
1020002	Chi (1)	Río Desaguadero Cotacotani	Altiplanicas	-18.21	-69.19	4500	1964-12 / 2016-10	544	15.3	
1020003	Chi (1)	Río Lauca En Estancia El Lago	Altiplanicas	-18.23	-69.33	4370	1937-2 / 2016-10	511	48.2	
1202001	Chi (1)	Río Lluta En Jamiraya	Río Lluta	-18.27	-69.67	2324	1937-11 / 1944-6	80	3.6	
1300002	Chi (1)	Río Laco En Cosapilla	Río San Jose	-18.39	-69.58	3000	1995-9 / 2016-10	243	7.3	
1300009	Chi (1)	Río Ticnamar En Angostura	Río San Jose	-18.51	-69.57	3010	2006-1 / 2017-6	110	22.5	
1021002	Chi (1)	Río Guallatire En Guallatire	Altiplanicas	-18.52	-69.17	4280	1971-6 / 2017-6	508	10.7	
1300001	Chi (1)	Río Ticnamar En Ticnamar	Río San Jose	-18.54	-69.54	3800	1994-12 / 2005-12	81	40.9	
1310003	Chi (1)	Río San Jose Antes Bocatoma Azapa	Río San Jose	-18.58	-69.97	960	1975-5 / 1984-12	98	18.3	
1021001	Chi (1)	Río Lauca En Japu (O En El Limite)	Altiplanicas	-18.59	-69.05	3907	1963-4 / 2016-10	420	36.6	
1041002	Chi (1)	Río Isluga En Bocatoma	Altiplanicas	-19.25	-68.78	4000	1995-6 / 2016-10	209	21.1	
1730004	Chi (1)	Quebrada Tarapaca En Mulli-Mulli	Pampa Del Tamarugal	-19.74	-69.02	3602	1931-4 / 1947-2	111	43.7	
1730007	Chi (1)	Quebrada Tarapaca En Sibaya	Pampa Del Tamarugal	-19.78	-69.14	2830	1928-9 / 2017-6	266	75.8	
1730002	Chi (1)	Río Tarapaca En Mina San Juan	Pampa Del Tamarugal	-19.83	-69.29	2113	1984-12 / 2003-12	203	13.6	
1730012	Chi (1)	Quebrada Tarapaca En Laonsana	Pampa Del Tamarugal	-19.86	-69.34	1847	2003-3 / 2014-4	97	29.7	
1044001	Chi (1)	Río Cancosa En El Tambo	Altiplanicas	-19.86	-68.59	4000	1994-8 / 2016-10	247	10.2	
1730003	Chi (1)	Río Coscaya En Saitoco	Pampa Del Tamarugal	-19.87	-68.93	3979	1985-5 / 2016-10	339	12.9	3 (None)
1730001	Chi (1)	Río Coscaya En Pampa Lirima	Pampa Del Tamarugal	-19.88	-68.95	3540	1977-12 / 1989-2	135	2.9	
1050003	Chi (1)	Río Batea En Confluencia	Altiplanicas	-20.03	-68.81	3956	1980-10 / 2016-10	390	12.6	
1050002	Chi (1)	Río Piga En Collacagua	Altiplanicas	-20.04	-68.80	3968	1959-11 / 2016-10	594	15.7	
1050004	Chi (1)	Río Collacagua En Peablanca	Altiplanicas	-20.13	-68.85	3853	1981-2 / 2016-10	379	14.3	
2113001	Chi (1)	Río Huatacondo En Copaquire	Río Loa	-20.93	-68.89	3540	1977-12 / 2016-6	418	12.4	
Bol01	Bol (3)	Puente Aruma (#)	Pilcomayo	-21.26	-63.51	400	2006-1 / 2016-12	105	19.2	

Bol02	Bol (3)	Villamontes (#)	Pilcomayo	-21.26	-63.51	390	1973-8 / 2016-10	487	6.5	
2101001	Chi (1)	Río Loa Antes Represa Lequena	Río Loa	-21.66	-68.66	3315	1967-7 / 2017-6	513	17.0	4 (7)
2103003	Chi (1)	Río San Pedro En Camino Internacional	Río Loa	-21.94	-68.54	3320	1979-10 / 1989-3	94	19.7	
2103002	Chi (1)	Río San Pedro En Parshall N"2 (Bt. Chilex)	Río Loa	-21.95	-68.51	3318	1967-11 / 2015-12	495	16.9	
2103001	Chi (1)	Río San Pedro En Parshall N"1	Río Loa	-21.97	-68.37	3700	1968-1 / 2016-10	468	22.5	
2104013	Chi (1)	Río Loa En Vado Santa Barbara (Doh)	Río Loa	-21.97	-68.61	2950	2006-4 / 2017-6	111	20.1	
2104003	Chi (1)	Río Loa En Alcantarilla Conchi N"2	Río Loa	-21.98	-68.61	2932	1979-9 / 2006-3	309	5.8	
2104001	Chi (1)	Río Loa En Conchi	Río Loa	-21.99	-68.61	3010	1916-10 / 1975-11	291	60.2	
2103014	Chi (1)	Río Siloli Antes B.T. Fcab	Río Loa	-22.01	-68.03	4000	2001-3 / 2017-6	185	8.4	
614	Arg (2)	Río Carapari - Puente Carretero	Río Itiyuro O Carapari	-22.11	-63.71	530	1942-9 / 1961-8	228	0.0	
607	Arg (2)	Río Bermejo - Alarache	Río Bermejo Superior	-22.26	-64.57	1050	1971-9 / 2016-7	400	25.8	
2105007	Chi (1)	Río Toconce Antes Represa Sendos	Río Loa	-22.27	-68.15	3362	1982-12 / 2016-4	332	19.6	
2105001	Chi (1)	Río Salado A. J. Curti	Río Loa	-22.28	-68.26	3080	1976-1 / 2016-10	452	10.5	
2105002	Chi (1)	Río Salado En Sifon Ayquina	Río Loa	-22.29	-68.34	2980	1975-4 / 2017-2	454	12.4	
638	Arg (2)	Río Tarija - Astilleros	Río Bermejo Superior	-22.36	-64.11	500	1968-9 / 1985-8	197	3.4	
2105005	Chi (1)	Río Salado Antes Junta Loa	Río Loa	-22.37	-68.65	2500	1916-10 / 2013-8	283	76.4	
631	Arg (2)	Río Pilcomayo - La Paz	Río Pilcomayo	-22.39	-62.51	230	1960-9 / 2016-8	672	0.0	5 (5)
605	Arg (2)	Río Bermejo - Balapuca	Río Bermejo Superior	-22.51	-64.45	530	1971-9 / 2016-8	540	0.0	
637	Arg (2)	Río Tarija - San Telmo	Río Bermejo Superior	-22.57	-64.24	500	1964-9 / 2016-8	430	31.1	
606	Arg (2)	Río Bermejo - Arrasayal	Río Bermejo Superior	-22.70	-64.40	500	1966-9 / 1986-8	224	6.7	
604	Arg (2)	Río Bermejo - Aguas Blancas	Río Bermejo Superior	-22.72	-64.37	405	1944-9 / 2016-8	864	0.0	
608	Arg (2)	Río Bermejo - Junta San Antonio	Río Bermejo Superior	-22.83	-64.29	344	1944-9 / 1960-8	192	0.0	
655	Arg (2)	Río Iruya - San Jose	Río Bermejo Superior	-22.89	-64.68	858	1980-2 / 2013-8	401	0.5	
681	Arg (2)	Río Astilleros - El Angosto	Río Bermejo Superior	-22.89	-64.68	840	1983-9 / 1988-8	60	0.0	
621	Arg (2)	Río Iruya - El Angosto	Río Bermejo Superior	-22.91	-64.58	500	1956-1 / 1979-9	138	51.6	
683	Arg (2)	Río Piedras - San Antonio	Río Bermejo Superior	-22.91	-64.63	800	1983-9 / 1992-8	108	0.0	
629	Arg (2)	Río Pescado - Cuatro Cedros	Río Bermejo Superior	-22.92	-64.42	450	1956-9 / 2016-8	706	1.9	
628	Arg (2)	Río Pescado - Colonia Colpana	Río Bermejo Superior	-22.96	-64.37	340	1945-9 / 1973-8	336	0.0	
641	Arg (2)	Río Blanco O Zenta - Vado Hondo	Río Bermejo Superior	-23.12	-64.50	400	1972-9 / 1980-1	74	16.9	
693	Arg (2)	Río Bermejo - Pozo Sarmiento	Río Bermejo Superior	-23.25	-64.14	296	1940-9 / 2016-8	900	1.3	6 (5)
2500002	Chi (1)	Canal Aguas Blancas	Salar De Atacama	-23.28	-67.95	2415	1977-1 / 2001-4	137	54.3	
2500005	Chi (1)	Canal Cuno En Socaire	Salar De Atacama	-23.62	-67.85	3600	1989-11 / 2016-10	306	8.4	

17	Arg (2)	Río Valle Grande - Peña Alta	Río San Francisco	-23.64	-64.96	1150	1952-9 / 1991-8	420	10.3	
16	Arg (2)	Río San Francisco – Caimancito (†)	Río San Francisco	-23.71	-64.54	367	1947-1 / 2016-8	764	8.6	7 (5)
2500003	Chi (1)	Canal Tulan En Tilomonte	Salar De Atacama	-23.78	-68.07	2450	1977-6 / 2000-8	110	61.7	
4	Arg (2)	Río Candelaria - Arrayanal	Río San Francisco	-23.93	-65.05	680	1967-9 / 1991-8	288	0.0	
18	Arg (2)	Río Yala - Los Nogales	Río San Francisco	-24.12	-65.40	1550	1953-9 / 1968-8	180	0.0	
15	Arg (2)	Río Reyes - Termas De Reyes	Río San Francisco	-24.17	-65.48	1750	1945-9 / 1960-11	147	19.7	
6	Arg (2)	Río Grande - Puente Perez	Río San Francisco	-24.18	-65.30	1250	1945-9 / 1954-8	80	25.9	
14	Arg (2)	Río Reyes - Antes Del Guerrero	Río San Francisco	-24.18	-65.43	1500	1945-9 / 1968-8	216	21.7	
7	Arg (2)	Río Guerrero - Puente Carretero	Río San Francisco	-24.19	-65.43	1500	1945-9 / 1968-8	240	13.0	
5	Arg (2)	Río Chico - Peña Blanca	Río San Francisco	-24.19	-65.29	1275	1945-9 / 1969-8	266	7.6	
10	Arg (2)	Río Los Alisos - Los Alisos	Río San Francisco	-24.26	-65.31	1690	1959-3 / 1968-5	96	13.5	
19	Arg (2)	Río Grande - San Juancito	Río San Francisco	-24.36	-65.00	900	1967-9 / 1981-5	159	3.6	
13	Arg (2)	Río Perico - El Tipal	Río San Francisco	-24.38	-65.33	1287	1927-9 / 1972-3	512	4.3	
619	Arg (2)	Río Dorado - Apolinario Saravia	Río Bermejo Medio E Inferior	-24.40	-63.99	358	1951-12 / 1967-8	187	1.1	
9	Arg (2)	Río Lavayen - Bajada Del Pinto	Río San Francisco	-24.43	-64.83	524	1942-9 / 1980-8	420	7.9	
8	Arg (2)	Río Las Maderas - El Angosto	Río San Francisco	-24.45	-65.25	1100	1930-9 / 1948-4	212	0.0	
635	Arg (2)	Río Santa Rufina - Santa Rufina	Río San Francisco	-24.56	-65.40	1560	1944-9 / 1955-8	132	0.0	
620	Arg (2)	Río Dorado - San Felipe/Sombrero	Río Bermejo Medio E Inferior	-24.56	-64.19	400	1967-9 / 1986-8	216	5.3	
623	Arg (2)	Río Las Nieves - El Volcan	Río San Francisco	-24.62	-65.46	1480	1947-12 / 1961-8	165	0.0	
640	Arg (2)	Río Yacones - Desembocadura Al Nieves	Río San Francisco	-24.62	-65.46	1480	1947-12 / 1961-8	165	0.0	
634	Arg (2)	Río San Alejo - San Alejo	Río San Francisco	-24.63	-65.45	1500	1944-9 / 1955-8	132	0.0	
636	Arg (2)	Río Seco - San Fernando	Río Bermejo Medio E Inferior	-24.65	-64.37	450	1967-9 / 1986-8	226	0.9	
617	Arg (2)	Río Del Valle - El Ceibal	Río Bermejo Medio E Inferior	-24.70	-64.19	480	1966-9 / 1986-8	236	1.7	
602	Arg (2)	Río Arenales - Salamanca	Alta Río Juramento	-24.71	-65.61	1770	1949-6 / 1962-4	154	0.6	
625	Arg (2)	Río Mojotoro - El Angosto	Río San Francisco	-24.71	-65.31	1070	1942-9 / 1986-8	484	8.3	
618	Arg (2)	Río Del Valle - El Piquete	Río Bermejo Medio E Inferior	-24.76	-64.47	520	1943-9 / 1973-8	360	0.0	
601	Arg (2)	Río Arenales - Potrero De Diaz	Alta Río Juramento	-24.82	-65.61	1249	1945-2 / 1967-8	271	0.0	
639	Arg (2)	Río Toro Y Blanco - Campo Quijano	Alta Río Juramento	-24.90	-65.67	1565	1930-1 / 1961-8	341	10.3	
610	Arg (2)	Río Blanco - Dique Nivelador	Alta Río Juramento	-24.90	-65.66	1590	1944-9 / 1961-8	204	0.0	
615	Arg (2)	Río Corralito - Peñas Bayas	Alta Río Juramento	-24.97	-65.75	1573	1943-9 / 1961-8	216	0.0	
603	Arg (2)	Río Arias - San Gabriel	Alta Río Juramento	-25.16	-65.42	1150	1941-10 / 1968-5	319	0.3	
686	Arg (2)	Río Juramento - Finca Agropecuaria	Río Pasaje O Salado	-25.16	-64.12	500	2001-10 / 2013-8	138	3.5	

626	Arg (2)	Río Juramento - El Tunal	Río Pasaje O Salado	-25.24	-64.37	425	1941-9 / 2013-8	594	31.3	
622	Arg (2)	Río Juramento - Cabra Corral	Río Pasaje O Salado	-25.27	-65.33	945	1934-9 / 1968-8	408	0.0	
695	Arg (2)	Río Juramento - El Quebrachal	Río Juramento	-25.29	-63.98	410	2001-10 / 2013-8	142	0.7	
624	Arg (2)	Río Medina - Desemb. Al Pasaje	Río Pasaje O Salado	-25.30	-64.54	470	1942-4 / 1986-8	533	0.0	
627	Arg (2)	Río Juramento - Miraflores	Río Pasaje O Salado	-25.35	-64.82	810	1929-1 / 1986-8	654	5.5	
612	Arg (2)	Río Calchaqui - Las Flechas	Alta Río Juramento	-25.68	-66.09	1950	1946-9 / 1954-8	96	0.0	
613	Arg (2)	Río Calchaqui - Dique Los Sauces	Alta Río Juramento	-25.79	-65.96	1850	1931-1 / 1959-9	323	6.4	
633	Arg (2)	Río Rosario - Toma De Ovando	Rios Rosario U Horcones Y Urueña	-25.80	-65.10	800	1948-9 / 1967-8	228	0.0	
632	Arg (2)	Río Pucara - El Angosto	Alta Río Juramento	-25.86	-66.31	2200	1940-9 / 1963-8	276	0.0	
611	Arg (2)	Río De Las Conchas - La Punilla	Alta Río Juramento	-26.00	-65.83	1790	1948-9 / 1968-6	238	0.0	
419	Arg (2)	Río Tala - El Brete	Río Sali-Dulce	-26.06	-65.35	845	1941-9 / 1962-8	252	0.0	
616	Arg (2)	Río Chuscha - Cafayate	Alta Río Juramento	-26.07	-65.98	1750	1941-9 / 1951-8	120	0.0	
427	Arg (2)	Río Chulca - Chulca	Río Sali-Dulce	-26.18	-65.51	1100	1953-2 / 1962-12	116	2.5	
420	Arg (2)	Río Urueña - 7 De Abril	Rios Rosario U Horcones Y Urueña	-26.22	-64.54	438	1948-10 / 1956-5	71	22.8	
426	Arg (2)	Río Tacanas - El Seminario	Río Sali-Dulce	-26.24	-65.49	1150	1944-6 / 1962-12	100	55.2	
428	Arg (2)	Río Rearte - La Junta	Río Sali-Dulce	-26.39	-65.51	1100	1953-1 / 1965-12	148	5.1	
429	Arg (2)	Río Potrero - Lambedero Blanco	Río Sali-Dulce	-26.43	-65.55	1150	1953-1 / 1963-12	132	0.0	
421	Arg (2)	Río Vipos - Toma Obras Sanitarias	Río Sali-Dulce	-26.48	-65.36	800	1937-9 / 1968-8	201	46.0	
3022001	Chi (1)	Río La Ola En Vertedero	Endorreicas Entre Frontera Y Vertiente	-26.48	-69.06	3583	1986-6 / 2016-10	332	11.7	
417	Arg (2)	Río Sali - San Vicente	Río Sali-Dulce	-26.50	-65.21	580	1960-9 / 1978-11	219	0.0	
416	Arg (2)	Río Sali - El Cadillal	Río Sali-Dulce	-26.62	-65.19	545	1913-9 / 1962-1	581	0.0	
403	Arg (2)	Río Calera - El Sunchal	Río Sali-Dulce	-26.63	-65.05	600	1947-9 / 1963-2	163	12.4	
203	Arg (2)	Río Ampajango - Desarenador	Alta Río Juramento	-26.81	-66.07	2100	1948-9 / 1960-12	103	30.4	
411	Arg (2)	Río Lules - Pot. De Las Tablas	Río Sali-Dulce	-26.86	-65.43	950	1914-9 / 2016-8	1046	14.5	8 (None)
407	Arg (2)	Río La Quebradita - Tafi Del Valle	Río Sali-Dulce	-26.87	-65.71	2285	1944-3 / 1949-12	70	0.0	
402	Arg (2)	Río La Angostura - Rp 307 Km 52	Río Sali-Dulce	-26.94	-65.68	1820	1943-9 / 1977-3	403	0.0	
210	Arg (2)	Río Santa Maria - Pie De Medano	Alta Río Juramento	-27.00	-66.25	2210	1970-9 / 2013-8	180	65.1	
3041005	Chi (1)	Río Lamas En El Salto	Endorreicas Entre Frontera Y Vertiente	-27.08	-68.93	4130	1980-3 / 2016-10	406	10.4	
415	Arg (2)	Río Reales - Casa De Piedra	Río Sali-Dulce	-27.09	-65.77	1150	1951-9 / 1961-12	114	8.1	
413	Arg (2)	Río Nevado - Casa De Piedra	Río Sali-Dulce	-27.09	-65.77	1150	1956-9 / 1962-8	72	0.0	
410	Arg (2)	Río Los Sosa - Rp 307 Km 19	Río Sali-Dulce	-27.10	-65.60	700	1953-9 / 2013-8	702	2.5	
450	Arg (2)	Río Pueblo Viejo - Los Ahujones	Río Sali-Dulce	-27.12	-65.78	1000	1949-7 / 1961-12	88	41.3	

406	Arg (2)	Río Horqueta - Los Ahujones	Río Sali-Dulce	-27.13	-65.78	1000	1953-2 / 1961-12	102	4.7	
3041004	Chi (1)	Río Valle Ancho En La Barrera	Endorreicas Entre Frontera Y Vertiente	-27.13	-69.01	3800	1979-5 / 2016-4	229	50.0	
470	Arg (2)	Río Sali - Ruta Prov. N323	Río Sali-Dulce	-27.14	-65.26	400	2004-9 / 2016-8	144	0.0	
3041002	Chi (1)	Río Villalobos En Vertedero	Endorreicas Entre Frontera Y Vertiente	-27.21	-69.03	3850	1979-11 / 2016-4	362	19.7	
3041003	Chi (1)	Río Valle Ancho Antes Junta Barros Negros	Endorreicas Entre Frontera Y Vertiente	-27.27	-69.01	3950	1979-5 / 2016-10	257	44.6	
3041001	Chi (1)	Río Barros Negros Antes Junta Valle Ancho	Endorreicas Entre Frontera Y Vertiente	-27.27	-69.00	3965	1980-3 / 2016-10	256	43.5	9 (None)
422	Arg (2)	Arroyo Membrillo - Las Higueras	Río Sali-Dulce	-27.31	-65.70	430	1948-12 / 1986-8	300	33.8	
418	Arg (2)	Río Solco - Las Higueras	Río Sali-Dulce	-27.31	-65.69	460	1943-1 / 1986-8	494	5.7	
404	Arg (2)	Río Cochuna - Los Hornitos	Río Sali-Dulce	-27.32	-65.91	1000	1943-1 / 1985-8	457	10.7	
405	Arg (2)	Río Conventillo - La Angostura	Río Sali-Dulce	-27.34	-65.73	480	1944-1 / 1961-12	191	11.6	
408	Arg (2)	Río Las Cañas - Las Hachas	Río Sali-Dulce	-27.36	-65.85	600	1943-9 / 1969-8	312	0.0	
471	Arg (2)	Río Seco - Ruta Prov. N157	Río Sali-Dulce	-27.38	-65.30	400	2004-9 / 2016-8	144	0.0	
409	Arg (2)	Río Las Cañas - Potrero Del Clavillo	Río Sali-Dulce	-27.40	-65.97	1300	1943-9 / 2016-8	876	0.0	
206	Arg (2)	Río Del Campo - Cabo San Miguel	Río Sali-Dulce	-27.40	-65.98	1300	1953-2 / 2013-8	119	83.6	
209	Arg (2)	Río Potrero - Chacras - Cabo San Miguel	Río Sali-Dulce	-27.40	-65.99	1300	1953-9 / 1961-12	100	0.0	
472	Arg (2)	Río Gastona - Ruta Prov. N157 - Atahona	Río Sali-Dulce	-27.46	-65.27	400	2004-9 / 2016-8	144	0.0	
3050001	Chi (1)	Río Astaburuaga En Cono	Endorreicas Entre Frontera Y Vertiente	-27.46	-69.05	4130	1979-12 / 2016-4	222	50.7	
473	Arg (2)	Río Chico - Ruta Prov. N157 - Monteagudo	Río Sali-Dulce	-27.51	-65.28	400	2004-9 / 2016-8	144	0.0	
204	Arg (2)	Río Andalgala - Andalgala	Salar De Pipanaco	-27.58	-66.31	959	1920-9 / 1960-12	436	9.9	
205	Arg (2)	Río Belen - Playa Larga	Salar De Pipanaco	-27.58	-67.00	1250	1946-9 / 1984-8	440	3.5	
474	Arg (2)	Río Marapa - Ruta Prov.N157 - Lamadrid	Río Sali-Dulce	-27.65	-65.24	450	2004-9 / 2016-8	144	0.0	
412	Arg (2)	Río Marapa - Embalse Escaba	Río Sali-Dulce	-27.66	-65.76	565	1938-9 / 1976-5	453	0.0	
423	Arg (2)	Río Singuil - Escaba	Río Sali-Dulce	-27.66	-65.77	600	1938-9 / 1957-8	228	0.0	
213	Arg (2)	Río San Ignacio - Barro Negro	Río Sali-Dulce	-27.71	-65.60	600	1949-1 / 1961-8	116	23.7	
3430003	Chi (1)	Río Copiapo En Pastillo	Río Copiapo	-28.00	-69.98	1300	1927-10 / 2017-6	716	35.4	10 (4)
201	Arg (2)	Río Abaucan - Tinosgasta	Río Abaucan	-28.04	-67.57	1200	1919-9 / 2013-8	487	56.8	
3404001	Chi (1)	Río Jorquera En Vertedero	Río Copiapo	-28.05	-69.96	1250	1949-4 / 2016-10	628	24.9	
3414001	Chi (1)	Río Pulido En Vertedero	Río Copiapo	-28.09	-69.94	1310	1964-2 / 2017-6	636	3.6	
3421001	Chi (1)	Río Manflas En Vertedero	Río Copiapo	-28.15	-69.99	1550	1964-2 / 2016-4	578	10.5	
207	Arg (2)	Río Del Valle - Pomancillo	Falda Oriental De Ambato	-28.36	-65.71	665	1917-9 / 1960-8	516	0.0	
211	Arg (2)	Río Tala - La Reja	Falda Oriental De Ambato	-28.55	-65.80	600	1936-9 / 1960-12	292	0.0	
1002	Arg (2)	Río Chañarmuyo - Chañarmuyo	Río Abaucan	-28.61	-67.58	1750	1967-9 / 1981-12	172	0.0	

202	Arg (2)	Río Albigasta - El Sauce D. Sotomayor	Salinas Grandes	-28.65	-65.14	330	1939-9 / 1962-8	276	0.0	
3820001	Chi (1)	Río Huasco En Algodones	Río Huasco	-28.73	-70.51	750	1975-7 / 2016-10	426	16.6	
1001	Arg (2)	Río Vinchina - Vinchina	Río Vinchina - Bermejo	-28.75	-68.22	1480	1966-9 / 1981-8	180	0.0	
3815001	Chi (1)	Río Carmen En Ramadillas	Río Huasco	-28.75	-70.48	825	1918-11 / 2016-11	733	39.6	
3806001	Chi (1)	Río Transito Antes Junta Río Carmen	Río Huasco	-28.76	-70.48	812	1927-10 / 2015-2	968	10.5	11 (4)
1004	Arg (2)	Río Famatina - Famatina	Cuenca Varias De Velazco	-28.92	-67.53	1100	1940-9 / 1982-8	504	0.0	
3815002	Chi (1)	Río Carmen En Pte. La Majada	Río Huasco	-28.93	-70.46	1075	1988-10 / 2016-10	264	23.9	
3814001	Chi (1)	Río Carmen En San Felix	Río Huasco	-28.94	-70.46	812	1929-12 / 1988-1	288	60.0	
3804002	Chi (1)	Río Transito En Angostura Pinte	Río Huasco	-28.94	-70.26	1000	1965-1 / 2016-10	594	7.3	
3802001	Chi (1)	Río Conay En Las Lozas	Río Huasco	-28.95	-70.10	1568	1984-11 / 2017-6	354	12.4	
3804001	Chi (1)	Río Transito En Los Tambos	Río Huasco	-28.97	-70.23	1400	1958-11 / 1971-12	141	13.5	
3814003	Chi (1)	Río Carmen En El Corral	Río Huasco	-29.11	-70.42	2000	1991-10 / 2017-6	305	4.1	
1003	Arg (2)	Río Durazno - Chilecito	Cuenca Varias De Velazco	-29.15	-67.49	1350	1940-9 / 1982-8	337	33.1	
1006	Arg (2)	Río Huaco - Dique Los Indios	Río Abaucan	-29.16	-67.08	1325	1971-9 / 1981-12	124	0.0	
1008	Arg (2)	Río La Rioja - Toma Sanagasta	Río Abaucan	-29.27	-67.02	1012	1923-3 / 1962-2	424	9.4	
1005	Arg (2)	Río Miranda - Miranda	Cuenca Varias De Velazco	-29.35	-67.71	1500	1967-9 / 1981-8	166	1.2	
9999	Arg (4)	Río Jáchal - Piedra Pintada	Río Jachal	-29.81	-69.16	1920	2001-3 / 2015-10	171	2.8	
4306001	Chi (1)	Río Turbio En Huanta	Río Elqui	-29.85	-70.39	1195	1928-11 / 1983-3	281	58.2	
4308001	Chi (1)	Río Turbio En Varillar	Río Elqui	-29.95	-70.54	860	1914-4 / 2017-3	1211	4.9	12 (4)
4302001	Chi (1)	Río Toro Antes Junta Río La Laguna	Río Elqui	-29.97	-70.09	2108	1985-6 / 2017-6	367	7.3	
4314002	Chi (1)	Río Claro En Rivadavia	Río Elqui	-29.98	-70.56	820	1914-3 / 2017-6	1168	8.5	
4320001	Chi (1)	Río Elqui En Algarrobal	Río Elqui	-30.00	-70.59	760	1916-9 / 2017-6	999	19.8	
4314001	Chi (1)	Río Claro En Montegrande	Río Elqui	-30.09	-70.49	1120	1947-2 / 1983-3	426	4.7	
4313001	Chi (1)	Río Cochiguaz En El Peñon	Río Elqui	-30.13	-70.44	1360	1983-8 / 2016-10	400	2.7	
1204	Arg (2)	Río Jachal - Pachimoco	Río Jachal	-30.20	-68.83	1160	1921-7 / 1990-6	732	11.6	
4301002	Chi (1)	Río La Laguna En Salida Embalse La Laguna	Río Elqui	-30.20	-70.04	3130	1928-11 / 2016-10	640	41.2	
1203	Arg (2)	Río Jachal - Las Trancas	Río Jachal	-30.20	-68.87	1350	1952-7 / 1964-6	144	0.0	
4311001	Chi (1)	Estero Derecho En Alcohuaz	Río Elqui	-30.22	-70.50	1645	1983-11 / 2016-10	399	2.2	
4502001	Chi (1)	Río Hurtado En La Cortadera	Río Limari	-30.34	-70.76	900	1968-2 / 1983-2	147	21.4	
4501002	Chi (1)	Río Hurtado En Las Breas	Río Limari	-30.39	-70.60	1645	1928-6 / 1977-11	380	37.9	
4503001	Chi (1)	Río Hurtado En Angostura De Pangue	Río Limari	-30.44	-71.00	485	1918-9 / 2016-10	1099	9.5	13 (4)
4501001	Chi (1)	Río Hurtado En San Agustin	Río Limari	-30.46	-70.54	2035	1963-1 / 2016-7	617	6.8	

4506002	Chi (1)	Río Hurtado En Entrada Embalse Recoleta	Río Limari	-30.48	-71.07	410	1928-6 / 1984-1	259	62.4	
4523002	Chi (1)	Río Grande En Puntilla San Juan	Río Limari	-30.71	-70.92	420	1942-3 / 2017-6	863	7.3	
4523001	Chi (1)	Río Grande En Agua Chica	Río Limari	-30.71	-70.90	440	1946-9 / 1983-2	420	6.9	
4522002	Chi (1)	Río Rapel En Junta	Río Limari	-30.71	-70.87	485	1959-4 / 2016-7	624	12.0	
4522001	Chi (1)	Río Rapel En Paloma	Río Limari	-30.74	-70.62	1190	1941-10 / 1983-3	192	62.6	
4520001	Chi (1)	Río Los Molles En Ojos De Agua	Río Limari	-30.75	-70.44	2355	1970-5 / 2016-7	539	5.8	
4516001	Chi (1)	Río Grande En Coipo	Río Limari	-30.78	-70.82	575	1942-12 / 1978-4	313	28.5	
4514001	Chi (1)	Río Mostazal En Cuestecita	Río Limari	-30.81	-70.61	1250	1969-10 / 2016-7	544	6.0	
4515002	Chi (1)	Río Mostazal En Caren	Río Limari	-30.84	-70.77	700	1972-8 / 2016-7	444	18.4	
4515001	Chi (1)	Río Mostazal Antes Junta Río Tulahuencito	Río Limari	-30.85	-70.71	685	1959-5 / 1967-10	101	3.8	
1009	Arg (2)	Río Portezuelo - Dique Malanzan	Pampa De Las Salinas	-30.86	-66.72	794	1932-9 / 1960-6	333	0.3	
1007	Arg (2)	Río Anzulon - Dique Anzulon	Salinas Grandes	-30.86	-66.27	550	1931-9 / 1985-3	503	21.8	
4513001	Chi (1)	Río Grande En Cuyano	Río Limari	-30.92	-70.78	870	1959-5 / 2016-10	680	4.4	
1202	Arg (2)	Río Castaño - Castaño Nuevo	Río San Juan	-30.97	-69.56	1650	1951-4 / 1987-8	426	2.5	
4511002	Chi (1)	Río Grande En Las Ramadas	Río Limari	-31.01	-70.58	1380	1961-5 / 2017-6	632	8.9	
4512001	Chi (1)	Río Tascadero En Desembocadura	Río Limari	-31.02	-70.67	1370	1962-11 / 2016-10	628	6.0	
4531002	Chi (1)	Río Cogoti Entrada Embalse Cogoti	Río Limari	-31.03	-71.05	670	1953-1 / 2017-6	623	21.8	
4534001	Chi (1)	Río Pama Entrada Embalse Cogoti	Río Limari	-31.08	-71.07	680	1953-1 / 1983-4	262	30.1	
4531001	Chi (1)	Río Cogoti En Cogoti 18	Río Limari	-31.09	-70.96	810	1942-1 / 1983-3	331	35.1	
4530001	Chi (1)	Río Cogoti En Fraguita	Río Limari	-31.12	-70.89	1065	1971-9 / 2017-4	505	10.5	
1211	Arg (2)	Río San Juan - Km 101	Río San Juan	-31.25	-69.18	1245	1971-7 / 2016-6	436	19.3	
4533002	Chi (1)	Río Pama En Valle Hermoso	Río Limari	-31.27	-70.99	850	1987-11 / 2016-10	329	8.1	
1209	Arg (2)	Río San Juan - Dique I. De La Roza	Río San Juan	-31.51	-68.63	715	1909-7 / 1989-6	959	0.1	
4721001	Chi (1)	Río Illapel En Las Burras	Río Choapa	-31.51	-70.81	1079	1962-3 / 2017-6	631	7.7	
1208	Arg (2)	Río San Juan - Km 47.3	Río San Juan	-31.52	-68.94	945	1909-7 / 2013-2	1239	0.4	14 (1)
4723001	Chi (1)	Río Illapel En Huintil	Río Choapa	-31.57	-70.97	775	1927-5 / 2017-6	870	22.0	
4712001	Chi (1)	Río Chalinga En La Palmilla	Río Choapa	-31.70	-70.71	800	1991-11 / 2017-6	308	2.8	
4713001	Chi (1)	Río Chalinga En San Agustin	Río Choapa	-31.72	-70.84	850	1928-11 / 1973-5	448	18.7	
4713002	Chi (1)	Río Chalinga En Potrero Maitenes	Río Choapa	-31.74	-70.93	638	1929-11 / 1952-11	264	7.7	
4704002	Chi (1)	Río Cuncumen Antes Bocatoma De Canales	Río Choapa	-31.83	-70.60	1360	1965-10 / 2005-11	348	29.8	
1206	Arg (2)	Río De Los Patos - La Plateada	Río San Juan	-31.86	-69.66	1870	1909-7 / 2016-6	1214	5.5	
4704001	Chi (1)	Río Cuncumen Antes Junta Choapa (Chacay)	Río Choapa	-31.87	-70.61	1030	2001-6 / 2017-6	192	3.0	

1201	Arg (2)	Río Blanco - El Horcajo	Río San Juan	-31.90	-69.70	1925	1950-7 / 2000-6	584	2.7	
1205	Arg (2)	Río De Los Patos - Alvarez Condarco	Río San Juan	-31.92	-69.71	1930	1950-9 / 2016-6	772	2.3	
4703002	Chi (1)	Río Choapa En Cuncumen	Río Choapa	-31.97	-70.59	1200	1965-10 / 2017-6	596	6.7	15 (1)
4703001	Chi (1)	Río Choapa Sobre El Río Valle	Río Choapa	-32.00	-70.58	1260	1965-12 / 1982-11	150	28.6	
4700001	Chi (1)	Estero El Soldado En Laguna El Pelado	Río Choapa	-32.01	-70.32	3500	1950-2 / 1985-2	109	74.9	
5101001	Chi (1)	Río Pedernal En Tejada	Río Petorca	-32.08	-70.76	1080	1963-1 / 2016-10	480	27.8	
5100001	Chi (1)	Río Sobrante En Piadero	Río Petorca	-32.23	-70.71	1300	1928-8 / 2017-6	653	40.6	16 (none)
5200001	Chi (1)	Río Alicahue En Colliguay	Río Ligua	-32.33	-70.74	780	1963-12 / 2017-6	602	9.1	
5414001	Chi (1)	Río Putaendo En Resguardo Los Patos	Río Aconcagua	-32.50	-70.58	1218	1939-9 / 2017-6	893	7.2	
1429	Arg (2)	Río Colorado - Punta De Vacas	Río Mendoza	-32.83	-69.70	2300	1977-1 / 2008-2	325	13.1	
1414	Arg (2)	Río Mendoza - Punta De Vacas	Río Mendoza	-32.85	-69.75	2450	1940-7 / 2000-6	587	18.5	
1421	Arg (2)	Río Vacas - Punta De Vacas	Río Mendoza	-32.85	-69.75	2405	1949-1 / 2016-6	766	5.4	
5410002	Chi (1)	Río Aconcagua En Chacabuquito	Río Aconcagua	-32.85	-70.51	950	1936-9 / 2017-6	950	4.9	17 (1)
5406001	Chi (1)	Río Colorado En Colorado	Río Aconcagua	-32.86	-70.42	1062	1964-11 / 2016-10	552	14.2	
5403003	Chi (1)	Río Aconcagua En Los Quilos	Río Aconcagua	-32.86	-70.42	1062	1965-2 / 1981-3	174	13.0	
5401003	Chi (1)	Río Juncal En Juncal	Río Aconcagua	-32.87	-70.17	1800	1913-2 / 2017-6	706	45.3	
1407	Arg (2)	Río Cuevas - Punta De Vacas	Río Mendoza	-32.87	-69.77	2415	1949-1 / 2016-6	758	6.4	
1420	Arg (2)	Río Tupungato - Punta De Vacas	Río Mendoza	-32.88	-69.77	2430	1949-1 / 2016-6	757	6.5	
5403002	Chi (1)	Río Aconcagua En Río Blanco	Río Aconcagua	-32.91	-70.30	1420	1970-4 / 2017-6	519	11.1	
5402001	Chi (1)	Río Blanco En Río Blanco	Río Aconcagua	-32.91	-70.30	1420	1914-1 / 2017-6	729	43.0	
1413	Arg (2)	Río Mendoza - Guido	Río Mendoza	-32.92	-69.24	1405	1956-7 / 2016-6	720	0.0	18 (1)
5411001	Chi (1)	Estero Pocuro En El Sifon	Río Aconcagua	-32.92	-70.54	1000	1930-12 / 2016-11	898	15.5	
1412	Arg (2)	Río Mendoza - Cacheuta	Río Mendoza	-33.02	-69.12	1250	1909-7 / 1990-6	972	0.0	
5722001	Chi (1)	Estero Arrayan En La Montosa	Río Maipo	-33.33	-70.46	880	1952-10 / 2017-6	651	18.6	
5721001	Chi (1)	Estero Yerba Loca Antes Junta San Francisco	Río Maipo	-33.34	-70.36	1350	1986-11 / 2017-6	370	2.4	
5722002	Chi (1)	Río Mapocho En Los Almendros	Río Maipo	-33.37	-70.45	966	1948-8 / 2016-10	755	10.5	
5730008	Chi (1)	Quebrada Ramon En Recinto Emos	Río Maipo	-33.43	-70.51	845	1991-2 / 2016-1	285	7.8	
5705001	Chi (1)	Río Colorado Antes Junta Río Olivares	Río Maipo	-33.49	-70.13	1500	1977-4 / 2017-6	407	18.1	
5706001	Chi (1)	Río Olivares Antes Junta Río Colorado	Río Maipo	-33.49	-70.14	1500	1977-2 / 2017-6	459	8.0	
5707002	Chi (1)	Río Colorado Antes Junta Río Maipo	Río Maipo	-33.59	-70.37	890	1940-8 / 2017-6	863	9.3	
5710001	Chi (1)	Río Maipo En El Manzano	Río Maipo	-33.60	-70.38	850	1946-11 / 2017-6	816	6.5	19 (1)
5703006	Chi (1)	Estero Glaciar Echaurren	Río Maipo	-33.61	-70.12	3000	1976-2 / 2004-3	128	63.3	

5703002	Chi (1)	Río Yeso En Embalse El Yeso	Río Maipo	-33.67	-70.09	2475	1945-10 / 1978-7	216	46.8	
5704002	Chi (1)	Río Maipo En San Alfonso	Río Maipo	-33.74	-70.30	1092	1942-3 / 2017-2	836	9.8	
5703004	Chi (1)	Río Yeso En Manzanito	Río Maipo	-33.75	-70.16	1677	1943-12 / 1951-6	93	0.0	
1419	Arg (2)	Río Tunuyan - Valle De Uco	Río Tunuyan	-33.78	-69.27	1200	1954-7 / 2016-6	744	0.0	20 (1)
5703003	Chi (1)	Río Yeso Antes Junta Río Maipo	Río Maipo	-33.79	-70.21	1250	1913-11 / 1958-8	399	28.0	
5702001	Chi (1)	Río Volcan En Queltehues	Río Maipo	-33.81	-70.22	1365	1914-11 / 2017-6	1022	19.5	
5701002	Chi (1)	Río Maipo En Las Melosas	Río Maipo	-33.85	-70.20	1527	1962-2 / 2015-10	325	51.1	
5701001	Chi (1)	Río Maipo En Las Hualtatas	Río Maipo	-33.98	-70.15	1820	1979-3 / 2012-12	382	8.8	
6006001	Chi (1)	Río Pangal En Pangal	Río Rapel	-34.25	-70.33	1500	1985-11 / 2017-6	347	11.3	
6008005	Chi (1)	Río Cachapoal En Pte Termas De Cauquenes	Río Rapel	-34.25	-70.57	700	2002-10 / 2016-11	166	5.1	
6003001	Chi (1)	Río Cachapoal 5 Km. Aguas Abajo Junta Cortaderal	Río Rapel	-34.35	-70.38	1127	1989-7 / 2016-11	275	18.9	
6000003	Chi (1)	Río Las Leas Ante Junta Río Cachapoal	Río Rapel	-34.36	-70.31	1296	2006-12 / 2017-6	115	11.5	
6002001	Chi (1)	Río Cortaderal Ante Junta Río Cachapoal	Río Rapel	-34.37	-70.33	1200	1985-9 / 2016-10	170	55.8	
6013001	Chi (1)	Río Claro En Hacienda Las Nieves	Río Rapel	-34.50	-70.70	720	1920-12 / 2017-6	722	39.5	
1409	Arg (2)	Río Diamante - Los Reyunos	Río Diamante	-34.61	-68.63	850	1917-7 / 1977-6	564	21.7	
1423	Arg (2)	Río Diamante - La Jaula	Río Diamante	-34.67	-69.32	1500	1971-1 / 2016-6	545	0.2	21 (1)
6027001	Chi (1)	Río Claro En El Valle	Río Rapel	-34.69	-70.88	476	1970-5 / 2017-6	533	8.6	
6028001	Chi (1)	Río Tinguiririca Bajo Los Briones	Río Rapel	-34.72	-70.83	560	1921-11 / 2017-6	888	24.9	22 (None)
6033001	Chi (1)	Estero Chimbarongo En Convento Viejo	Río Rapel	-34.77	-71.16	245	1968-11 / 1993-12	286	8.0	
6025001	Chi (1)	Río Tinguiririca Aguas Abajo Junta Río Azufre	Río Rapel	-34.82	-70.57	1024	1970-5 / 1993-4	195	31.6	
7104001	Chi (1)	Estero El Manzano Antes Junta Río Teno	Río Mataquito	-34.97	-70.94	690	1959-8 / 1984-10	288	7.7	
7104002	Chi (1)	Río Teno Despues De Junta Con Claro	Río Mataquito	-35.00	-70.82	647	1948-6 / 2017-6	711	16.7	
7102001	Chi (1)	Río Teno En Los Queñes	Río Mataquito	-35.00	-70.81	900	1938-4 / 1985-1	557	4.0	
7103001	Chi (1)	Río Claro En Los Queñes	Río Mataquito	-35.00	-70.81	900	1929-5 / 2016-8	1047	3.1	23 (2)
7102005	Chi (1)	Río Teno Bajo Quebrada Infiernillo	Río Mataquito	-35.05	-70.64	990	1985-1 / 2016-2	285	26.0	
1428	Arg (2)	Río Atuel - El Sosneado	Río Atuel	-35.08	-69.61	1595	1972-7 / 2016-6	521	1.3	
1403	Arg (2)	Río Atuel - La Angostura	Río Atuel	-35.09	-68.88	1200	1906-7 / 2016-6	1320	0.0	24 (1)
1406	Arg (2)	Río Cobre - Valle Hermoso	Río Colorado	-35.18	-70.25	2150	1918-7 / 1970-6	576	7.7	
7116001	Chi (1)	Estero Upeo En Upeo	Río Mataquito	-35.18	-71.09	450	1963-2 / 2016-11	603	9.3	
1415	Arg (2)	Río Salado - Cañada Ancha	Río Atuel	-35.20	-69.76	1715	1940-7 / 2016-6	884	3.1	
1435	Arg (2)	Río Atuel - Loma Negra	Río Atuel	-35.27	-69.26	1360	1980-11 / 2016-6	428	0.0	

7112001	Chi (1)	Río Colorado En Junta Con Palos	Río Mataquito	-35.29	-70.99	600	1917-11 / 2017-2	743	39.5	
7115001	Chi (1)	Río Palos En Junta Con Colorado	Río Mataquito	-35.29	-70.99	600	1947-5 / 2017-6	605	30.2	
1434	Arg (2)	Río Grande - La Estrechura	Río Colorado	-35.32	-70.29	1980	1978-2 / 2015-6	378	15.8	
1447	Arg (2)	Río Valenzuela - Valle Noble	Río Colorado	-35.33	-70.30	2000	1977-11 / 2015-6	361	20.1	
1426	Arg (2)	Río Pincheira - Pincheira	Laguna De Llancanelo	-35.52	-69.81	1760	1968-7 / 2016-6	461	20.0	25 (1)
1454	Arg (2)	Río Malargüe - La Barda	Laguna De Llancanelo	-35.55	-69.68	1560	1987-7 / 2016-6	334	4.0	
7320003	Chi (1)	Río Claro En San Carlos	Río Maule	-35.70	-71.07	550	2002-11 / 2017-6	177	2.2	
1446	Arg (2)	Río Chico - Las Loicas	Río Colorado	-35.79	-70.15	1620	1992-5 / 2016-6	290	0.0	
7355003	Chi (1)	Río Ancoa Antes Tunel Canal Melado	Río Maule	-35.87	-71.12	800	1961-12 / 1995-4	232	43.8	
1425	Arg (2)	Río Poti Malal - Gendarmeria	Río Colorado	-35.87	-69.95	1460	1971-8 / 2016-6	524	2.8	
1427	Arg (2)	Río Grande - La Gotera	Río Colorado	-35.87	-69.89	1430	1972-1 / 2013-6	478	4.0	
7317005	Chi (1)	Río Melado En El Salto	Río Maule	-35.88	-71.02	730	2003-4 / 2017-6	172	2.3	
7354001	Chi (1)	Río Achibueno En Los Pegnascos	Río Maule	-35.96	-71.49	590	1947-3 / 1986-9	435	11.0	
7354002	Chi (1)	Río Achibueno En La Recova	Río Maule	-36.00	-71.44	309	1986-11 / 2017-6	360	5.0	
7350003	Chi (1)	Río Longavi En El Castillo	Río Maule	-36.25	-71.35	598	1964-5 / 2016-8	552	14.7	
7330001	Chi (1)	Río Perquilauquen En San Manuel	Río Maule	-36.38	-71.62	266	1930-9 / 2017-6	914	14.8	26 (2)
8106001	Chi (1)	Río Ñuble En San Fabian	Río Itata	-36.57	-71.56	410	1946-5 / 1998-11	611	6.1	
8106002	Chi (1)	Río Ñuble En San Fabian N 2	Río Itata	-36.59	-71.53	450	1999-3 / 2017-6	219	3.1	
8112001	Chi (1)	Río Niblinto Antes Canal Alimentador Emb. Coihueco	Río Itata	-36.65	-71.76	290	1971-2 / 2013-12	331	37.7	
8105001	Chi (1)	Río Ñuble En La Punilla	Río Itata	-36.66	-71.32	635	1957-4 / 2016-10	667	9.5	
8104001	Chi (1)	Río Sauces Antes Junta Con Ñuble	Río Itata	-36.67	-71.28	620	1966-11 / 2016-10	538	12.9	
8117004	Chi (1)	Río Chillan En Esperanza	Río Itata	-36.79	-71.75	435	1939-3 / 1994-8	668	2.6	
8117006	Chi (1)	Río Chillan En Esperanza N 2	Río Itata	-36.80	-71.66	575	2003-12 / 2017-6	149	11.3	
2001	Arg (2)	Río Barrancas - Barrancas	Río Colorado	-36.81	-69.89	950	1960-7 / 2015-6	574	13.0	
2077	Arg (2)	Río Neuquen - Varvarco	Río Neuquen	-36.86	-70.68	1180	1981-6 / 2004-3	206	24.8	
8130001	Chi (1)	Río Renegado En Invernada	Río Itata	-36.87	-71.63	710	1940-3 / 2015-12	836	10.9	
8130002	Chi (1)	Río Diguillin En San Lorenzo (Atacalco)	Río Itata	-36.93	-71.58	707	1946-6 / 2017-6	846	3.8	27 (2)
2002	Arg (2)	Río Colorado - Buta Ranquil	Río Colorado	-37.07	-69.75	850	1940-4 / 2016-6	915	0.0	28 (1)
8372002	Chi (1)	Río Polcura En Cuatro Junta	Río Bio-Bio	-37.11	-71.25	1319	2003-2 / 2017-6	166	6.7	
2034	Arg (2)	Río Nahueve - Los Carrizos	Río Neuquen	-37.12	-70.77	1150	1974-4 / 2004-3	357	0.8	
2036	Arg (2)	Río Neuquen - Andacollo	Río Neuquen	-37.18	-70.68	1000	1971-6 / 2004-3	379	3.8	

8380001	Chi (1)	Río Laja En Tucapel	Río Bio-Bio	-37.28	-71.99	285	1916-3 / 2015-2	818	33.2	
2012	Arg (2)	Río Curileuvu - Los Maitenes	Río Neuquen	-37.32	-70.28	959	1989-5 / 2004-3	179	0.0	
8375001	Chi (1)	Río Polcura A. Desc. Central El Toro	Río Bio-Bio	-37.33	-71.55	710	2003-8 / 2017-6	168	2.3	
8376001	Chi (1)	Río Rucue En Camino A Antuco	Río Bio-Bio	-37.34	-71.80	450	1983-11 / 2016-2	321	19.8	
2059	Arg (2)	Río Neuquen - Rahueco	Río Neuquen	-37.37	-70.35	879	1984-4 / 2008-2	275	4.2	
8323002	Chi (1)	Río Duqueco En Villucura	Río Bio-Bio	-37.55	-72.03	228	1941-1 / 2016-10	876	6.6	29 (2)
8317002	Chi (1)	Río Lirquen En Cerro El Padre	Río Bio-Bio	-37.78	-71.87	340	1942-12 / 2016-9	883	3.3	
8313000	Chi (1)	Río Pangue En Captacion	Río Bio-Bio	-37.90	-71.60	550	2002-12 / 2017-6	170	5.6	
8341001	Chi (1)	Río Renaico En Jauja	Río Bio-Bio	-38.03	-71.94	418	1948-4 / 1995-3	106	81.8	
8307002	Chi (1)	Río Bio-Bio En Llanquen	Río Bio-Bio	-38.20	-71.30	750	2003-2 / 2017-6	174	2.2	
8350001	Chi (1)	Río Malleco En La Laguna	Río Bio-Bio	-38.22	-71.83	850	1960-6 / 1984-3	197	33.0	
2010	Arg (2)	Río Agrio - Bajada Del Agrio	Río Neuquen	-38.37	-70.03	660	1953-4 / 2016-12	765	0.0	
9123001	Chi (1)	Río Cautin En Rari-Ruca	Río Imperial	-38.43	-72.01	425	1929-3 / 2017-6	1036	5.1	30 (2)
8304001	Chi (1)	Río Lonquimay Antes Junta Río Bio Bio	Río Bio-Bio	-38.43	-71.23	876	1985-4 / 2016-5	368	4.4	
9122002	Chi (1)	Río Blanco En Curacautin	Río Imperial	-38.44	-71.92	405	1986-10 / 2015-10	340	5.6	
2004	Arg (2)	Río Neuquen - Paso De Indios	Río Neuquen	-38.53	-69.41	498	1903-4 / 2016-12	1346	1.4	31 (2)
9126001	Chi (1)	Río Collin En Codahue	Río Imperial	-38.58	-72.19	250	1996-8 / 2015-9	197	16.9	
9131001	Chi (1)	Río Quepe En Vilcun	Río Imperial	-38.68	-72.23	292	1946-4 / 2015-4	741	13.3	
9402001	Chi (1)	Río Allipen En Melipeuco	Río Tolten	-38.87	-71.73	450	1985-1 / 2016-10	372	5.3	
9405001	Chi (1)	Río Curaco En Colico	Río Tolten	-39.05	-72.09	250	1986-11 / 2016-10	293	21.0	
9416001	Chi (1)	Río Liucura En Liucura	Río Tolten	-39.26	-71.82	402	1971-10 / 2016-11	481	13.8	
9420001	Chi (1)	Río Tolten En Villarica	Río Tolten	-39.27	-72.24	250	1929-3 / 2017-6	1022	6.4	32 (2)
9414001	Chi (1)	Río Trancura Antes Río Llafenco	Río Tolten	-39.33	-71.77	386	1970-10 / 2016-10	534	6.3	
9412001	Chi (1)	Río Trancura En Curarrehue	Río Tolten	-39.36	-71.58	400	1968-9 / 2017-6	569	5.6	
2060	Arg (2)	Río Alumine - La Siberia	Río Limay	-39.38	-70.93	1190	1978-10 / 2015-10	345	22.5	
10102001	Chi (1)	Río Liquine En Liquine	Río Valdivia	-39.73	-71.86	600	1986-7 / 2016-4	340	7.9	
10111001	Chi (1)	Río San Pedro En Desague Lago Rinihue	Río Valdivia	-39.76	-72.47	115	1929-1 / 2016-10	429	60.5	33 (2)
2032	Arg (2)	Río Malleo - Malleo	Río Limay	-39.78	-71.04	800	1973-5 / 2015-10	465	8.8	
2005	Arg (2)	Río Chimehuin - Naciente	Río Limay	-39.79	-71.20	875	1936-1 / 2015-8	862	9.8	
10100002	Chi (1)	Río Fui En Desage Lago Pirihueico	Río Valdivia	-39.88	-71.89	600	1926-3 / 2016-10	372	66.8	
10100001	Chi (1)	Río Huahum En Desembocadura Lago Pirihueico	Río Valdivia	-40.02	-71.71	598	1941-2 / 1948-2	85	2.3	
2040	Arg (2)	Río Quilquihue - Junin De Los Andes	Río Limay	-40.06	-71.09	750	1962-4 / 2015-12	618	4.2	

10100006	Chi (1)	Río Huahum En La Frontera	Río Valdivia	-40.10	-71.68	616	2002-7 / 2016-10	168	5.1	
10305001	Chi (1)	Río Caunahue Camino A Llifen	Río Bueno	-40.16	-72.25	90	1997-1 / 2016-9	229	6.1	
10304001	Chi (1)	Río Calcurrupe En Desembocadura	Río Bueno	-40.23	-72.25	160	1986-7 / 2016-10	354	5.6	
10306001	Chi (1)	Río Nilahue En Mayay	Río Bueno	-40.28	-72.18	80	1987-8 / 2017-6	362	2.2	
2007	Arg (2)	Río Filohuahum - Filohuahum	Río Limay	-40.48	-71.24	730	1903-4 / 1947-3	377	28.6	
1818	Arg (2)	Río Pichi Leufu - Paso Flores	Río Limay	-40.49	-70.61	575	1960-4 / 1969-3	72	33.3	
1804	Arg (2)	Río Limay - Paso Limay (‡)	Río Limay	-40.53	-70.43	538	1903-4 / 1990-3	1025	1.8	34 (2)
1803	Arg (2)	Río Limay - Paso Flores	Río Limay	-40.57	-70.64	566	1941-4 / 2015-12	626	30.2	
2021	Arg (2)	Río Cuyin Manzano - Cuyin Manzano	Río Limay	-40.73	-71.15	675	1971-5 / 2015-1	478	9.0	
10340001	Chi (1)	Río Rahue En Desague Lago Rupanco	Río Bueno	-40.79	-72.70	130	1986-2 / 2017-5	371	4.1	35 (None)
10343001	Chi (1)	Río Coihueco Antes Junta Pichicope	Río Bueno	-40.93	-72.70	150	1987-8 / 2016-10	341	5.8	
1802	Arg (2)	Río Limay - Nahuel Huapi	Río Limay	-41.06	-71.15	760	1921-4 / 2015-12	750	34.0	
10454001	Chi (1)	Río Petrohue En Desague Lago Todos Los Santos	Cuencas E Islas R. Bueno - R. Puelo	-41.14	-72.41	150	1940-2 / 1947-5	89	2.2	
1834	Arg (2)	Río Manso Superior - Mascardi	Rios Manso Y Puelo	-41.25	-71.66	800	1969-7 / 1991-3	261	0.0	
1835	Arg (2)	Río De Los Césares - Mascardi	Rios Manso Y Puelo	-41.27	-71.66	800	1970-4 / 1987-3	204	0.0	
10411003	Chi (1)	Río Maullin En Llanquihue	Cuencas E Islas R. Bueno - R. Puelo	-41.27	-73.01	50	1929-3 / 1946-11	215	2.3	
1807	Arg (2)	Río Manso - Los Moscos	Rios Manso Y Puelo	-41.35	-71.64	795	1946-5 / 2016-3	815	2.9	36 (None)
1806	Arg (2)	Río Manso - Los Alerces	Rios Manso Y Puelo	-41.37	-71.74	700	1951-4 / 2016-3	769	1.4	
1812	Arg (2)	Río Roca - Los Alerces	Rios Manso Y Puelo	-41.38	-71.76	711	1985-11 / 2016-3	355	2.7	
10431000	Chi (1)	Río Blanco Ante Junta Río Chamiza	Cuencas E Islas R. Bueno - R. Puelo	-41.44	-72.60	242	2002-10 / 2015-4	133	14.7	
1805	Arg (2)	Río Manso - Lago Steffen	Rios Manso Y Puelo	-41.51	-71.55	325	1956-4 / 2016-3	387	46.3	
1813	Arg (2)	Río Villegas - Ruta Nac. N258	Rios Manso Y Puelo	-41.58	-71.50	590	1953-4 / 2016-3	400	47.1	
1814	Arg (2)	Río Manso - Confluencia	Rios Manso Y Puelo	-41.59	-71.69	470	1965-5 / 2016-3	586	4.1	
1815	Arg (2)	Río Foyel - Confluencia Con El Manso	Rios Manso Y Puelo	-41.59	-71.68	500	1966-4 / 2013-3	144	74.5	
10520001	Chi (1)	Río Puelo En Desague Lago Tagua Tagua	Río Puelo	-41.64	-72.18	22	2002-6 / 2016-10	155	12.9	
10523002	Chi (1)	Río Puelo En Carrera Basilio	Río Puelo	-41.65	-72.30	6	2003-4 / 2017-6	172	2.3	
1831	Arg (2)	Río Escondido - El Foyel	Rios Manso Y Puelo	-41.68	-71.57	585	1977-4 / 2016-3	455	2.8	
1836	Arg (2)	Río Chico - Cerro Mesa	Río Chubut	-41.71	-70.48	747	1956-8 / 2016-3	693	3.2	
1816	Arg (2)	Río Foyel - Ruta Nac. N258	Rios Manso Y Puelo	-41.72	-71.45	660	1963-4 / 1995-3	132	65.6	
10503001	Chi (1)	Río Manso Antes Junta Río Puelo	Río Puelo	-41.73	-72.05	28	2001-6 / 2016-7	169	9.6	
10514001	Chi (1)	Río Puelo Antes Junta Con Manso	Río Puelo	-41.75	-72.08	23	2001-8 / 2016-8	170	8.6	
1810	Arg (2)	Río Ñorquinco - Fita Timen	Río Chubut	-41.80	-70.88	950	1956-8 / 1981-3	232	21.6	

2267	Arg (2)	Río Alto Chubut - Nacimiento	Río Chubut	-41.81	-71.13	575	1967-7 / 2016-3	428	26.8	
2278	Arg (2)	Río Ñorquinco - Ñorquinco	Río Chubut	-41.86	-70.90	751	1982-4 / 2016-3	392	3.9	
1811	Arg (2)	Río Quemquemtreu - Escuela N139	Rios Manso Y Puelo	-41.90	-71.51	409	1956-6 / 2016-3	711	1.0	
1870	Arg (2)	Río Raquel - El Azul	Rios Manso Y Puelo	-41.93	-71.56	390	1993-7 / 2016-3	272	0.4	
1817	Arg (2)	Río Azul - El Azul	Rios Manso Y Puelo	-41.94	-71.56	292	1970-4 / 2016-3	552	0.0	
1895	Arg (2)	Río Quemquemtreu - Quemquemtreu	Rios Manso Y Puelo	-41.95	-71.54	402	2009-4 / 2016-3	84	0.0	
2206	Arg (2)	Río Chubut - El Maitén	Río Chubut	-42.06	-71.15	680	1943-4 / 2016-3	870	0.7	
2240	Arg (2)	Río Catarata - Hoyo De Epuyén	Rios Manso Y Puelo	-42.06	-71.51	320	1968-4 / 1990-3	252	4.5	
2314	Arg (2)	Río Azul - Puesto 2	Rios Manso Y Puelo	-42.09	-71.62	209	2009-4 / 2016-3	84	0.0	
2315	Arg (2)	Río Puelo - Hito Fronterizo	Rios Manso Y Puelo	-42.10	-71.73	189	2009-4 / 2016-3	84	0.0	
2227	Arg (2)	Río Mercedes - Rn 258	Los Rios Manso Y Puelo	-42.16	-71.41	260	1959-4 / 1985-3	300	3.8	
2208	Arg (2)	Río Epuyén - La Angostura	Rios Manso Y Puelo	-42.19	-71.40	290	1951-4 / 2016-3	729	6.5	
10904001	Chi (1)	Río Grande En San Pedro	Islas Chiloe Y Circundantes	-42.38	-73.87	97	1990-8 / 2016-10	276	14.8	37 (None)
2245	Arg (2)	Río Chico - Fofo Cahuel	Río Chubut	-42.41	-70.48	510	1973-4 / 1984-3	96	27.3	
2204	Arg (2)	Río Carrileufú - Cholila	Río Futaleufu	-42.50	-71.54	532	1957-4 / 2016-3	694	2.0	38 (3)
2301	Arg (2)	Río Chubut - Gualjaina	Río Chubut	-42.63	-70.32	474	1990-8 / 2016-3	308	0.0	
2211	Arg (2)	Río Gualjaina - Gualjaina	Río Chubut	-42.65	-70.42	480	1956-7 / 2016-3	702	2.1	
2228	Arg (2)	Río Lepá - Gualjaina	Río Chubut	-42.73	-70.54	506	1956-7 / 2012-12	395	41.7	
2221	Arg (2)	Río Arrayanes - Lago Verde	Río Futaleufu	-42.73	-71.74	509	2009-4 / 2016-3	84	0.0	
2242	Arg (2)	Río Frey - Nacimiento	Río Futaleufu	-42.91	-71.73	480	1948-4 / 1995-3	480	14.9	
2230	Arg (2)	Río Cohihues - Parque Nac. Los Alerces	Río Futaleufu	-42.97	-71.58	618	1956-6 / 2016-3	636	11.4	
2214	Arg (2)	Río Percey - Ruta A Futalaufquen	Río Futaleufu	-42.99	-71.49	500	1956-4 / 1994-3	149	67.3	
2218	Arg (2)	Río Fontana - Estancia Amancay	Río Futaleufu	-42.99	-71.56	627	1956-7 / 2016-3	699	2.5	
2259	Arg (2)	Río Futaleufú - Embalse	Río Futaleufu	-43.11	-71.65	495	1976-4 / 2016-3	449	6.5	
2217	Arg (2)	Río Situación - Escuela N90	Río Futaleufu	-43.13	-71.56	349	1962-4 / 1990-3	300	10.7	
2210	Arg (2)	Río Futaleufú - Balsa Garzón	Río Futaleufu	-43.14	-71.61	320	1948-4 / 1976-3	336	0.0	
2213	Arg (2)	Río Nanty Fall - Ruta A Chile	Río Futaleufu	-43.17	-71.51	338	1956-4 / 1994-3	451	1.1	
2220	Arg (2)	Río Futaleufu - Puesto Rios	Río Futaleufu	-43.18	-71.66	331	2009-4 / 2016-3	84	0.0	
10702002	Chi (1)	Río Futaleufu En La Frontera	Río Yelcho	-43.19	-71.78	314	2001-7 / 2017-6	189	4.1	
2239	Arg (2)	Río Bagglits - Ruta A Chile	Río Futaleufu	-43.20	-71.58	375	1977-8 / 2016-3	449	3.2	
10701002	Chi (1)	Río Espolon En Desague Lago Espolon	Río Yelcho	-43.22	-71.94	380	2001-7 / 2016-8	166	11.2	
10704002	Chi (1)	Río Futaleufu Ante Junta Río Malito	Río Yelcho	-43.45	-72.11	42	2002-4 / 2016-10	157	12.8	

2226	Arg (2)	Río Hielo - Confluencia	Rios Carrenleufu Y Pico	-43.52	-71.61	339	1964-4 / 2016-3	398	36.2	
2201	Arg (2)	Río Carrenleufú - Carrenleufú	Rios Carrenleufu Y Pico	-43.53	-71.60	322	1964-4 / 2016-3	556	10.9	
2279	Arg (2)	Río Huemul - Corcovado	Rios Carrenleufu Y Pico	-43.54	-71.47	405	1990-4 / 2016-3	299	4.2	
2288	Arg (2)	Río Carrenleufú - Puente De Hierro	Rios Carrenleufu Y Pico	-43.54	-71.49	390	1990-8 / 2016-3	308	0.0	
2293	Arg (2)	Río Carrenleufú - Poncho Moro	Rios Carrenleufu Y Pico	-43.61	-71.42	468	1991-7 / 2016-3	297	0.0	
11020003	Chi (1)	Río Encuentro Despues De Confluencia	Río Palena Y Costeras Limite Decima Region	-43.62	-71.70	414	1959-1 / 1966-6	78	16.1	
11020002	Chi (1)	Río Encuentro Antes Junta Estero Lopez	Río Palena Y Costeras Limite Decima Region	-43.63	-71.70	450	1959-2 / 1966-6	83	9.8	
11020001	Chi (1)	Estero Lopez Antes Junta Río Encuentro	Río Palena Y Costeras Limite Decima Region	-43.63	-71.70	450	1959-2 / 1966-7	81	12.9	
2282	Arg (2)	Río Poncho Moro - Ruta Prov. N44	Rios Carrenleufu Y Pico	-43.67	-71.42	470	1990-4 / 2016-3	282	9.6	
2202	Arg (2)	Río Carrenleufú - La Elena	Rios Carrenleufu Y Pico	-43.69	-71.32	783	1954-4 / 2016-3	735	1.2	39 (3)
11020004	Chi (1)	Río Tigre En La Frontera	Río Palena Y Costeras Limite Decima Region	-43.72	-71.71	650	2001-7 / 2016-8	169	9.6	
2207	Arg (2)	Río Chubut – Los Altares	Río Chubut	-43.89	-68.40	275	1943-4 / 2016-6	879	0.0	40 (none)
2203	Arg (2)	Río Carrenleufú - Lago Vinter	Rios Carrenleufu Y Pico	-43.90	-71.41	940	1955-4 / 2016-3	387	47.1	
11040001	Chi (1)	Río Palena Bajo Junta Rosselot	Río Palena Y Costeras Limite Decima Region	-44.00	-72.43	40	1999-6 / 2017-6	210	5.8	
11130001	Chi (1)	Río Ventisquero En Carretera Austral	Costeras E Islas R. Palena - R. Aisen	-44.46	-72.56	45	1991-9 / 2016-8	237	23.3	
11141001	Chi (1)	Río Cisnes En Estancia Río Cisnes	Costeras E Islas R. Palena - R. Aisen	-44.60	-71.55	480	1984-12 / 2016-7	346	11.5	41 (3)
11147002	Chi (1)	Río Grande En Carretera Austral	Costeras E Islas R. Palena - R. Aisen	-44.64	-72.28	200	1991-9 / 2016-8	278	10.0	
11143001	Chi (1)	Río Cisnes Antes Junta Río Moro	Costeras E Islas R. Palena - R. Aisen	-44.66	-71.81	500	1988-9 / 2017-6	337	5.3	
11147001	Chi (1)	Río Cisnes En Puerto Cisnes	Costeras E Islas R. Palena - R. Aisen	-44.75	-72.70	7	2001-5 / 2016-3	170	7.6	
11143002	Chi (1)	Río Moro Antes Junta Río Cisnes	Costeras E Islas R. Palena - R. Aisen	-44.75	-72.71	516	1988-7 / 2016-8	308	11.5	
2215	Arg (2)	Río Senguerr – Nacimiento (‡)	Rios Senguerr Y Chico	-44.96	-71.34	940	1949-4 / 2016-3	676	15.9	42 (3)
11302001	Chi (1)	Río Ñireguao En Villa Mañiguales	Río Aisen	-45.17	-72.14	188	1980-6 / 2016-8	406	9.4	
11307001	Chi (1)	Río Emperador Guillermo Antes Junta Mañiguales	Río Aisen	-45.26	-72.27	180	1980-6 / 2016-8	379	15.4	
11342001	Chi (1)	Río Aysen En Puerto Aysen	Río Aisen	-45.41	-72.64	10	1996-1 / 2017-6	250	5.7	
11337001	Chi (1)	Río Blanco Antes Junta Río Aysen	Río Aisen	-45.41	-72.61	1250	1998-11 / 2017-6	209	9.1	
2216	Arg (2)	Río Senguerr - Vuelta Del Senguerr	Rios Senguerr Y Chico	-45.47	-69.83	292	1937-4 / 1958-5	254	0.0	
11405001	Chi (1)	Río Lagunillas En Desague Lago Condor	Costeras E Islas R Aisen R Baker C. Gral. Martinez	-45.47	-72.91	10	1985-8 / 2013-7	260	24.9	
11317001	Chi (1)	Río Simpson Bajo Junta Coyhaique	Río Aisen	-45.55	-72.07	210	1969-4 / 2017-6	385	35.4	
11316001	Chi (1)	Río Coyhaique En Tejas Verdes	Río Aisen	-45.57	-72.04	335	1979-11 / 2016-8	320	29.7	
11315001	Chi (1)	Río Claro En Piscicultura	Río Aisen	-45.57	-72.08	900	1984-5 / 2016-8	183	54.3	
2264	Arg (2)	Río Senguerr - Puente Camino Buen Pasto	Rios Senguerr Y Chico	-45.64	-69.19	264	1946-1 / 2013-3	419	48.1	
2212	Arg (2)	Río Mayo - Paso Río Mayo	Rios Senguerr Y Chico	-45.68	-70.26	425	1945-1 / 2016-3	682	20.2	

11314001	Chi (1)	Río Pollux Antes Junta Simpson	Río Aisen	-45.70	-72.14	340	1979-12 / 1987-5	91	1.1	
11335002	Chi (1)	Río Blanco En Desague Lago Caro	Río Aisen	-45.78	-72.62	270	1985-3 / 2016-8	304	21.9	
11312001	Chi (1)	Río Blanco Antes Junta Huemules	Río Aisen	-45.81	-71.92	450	1979-11 / 2001-2	223	15.2	
11310001	Chi (1)	Río Huemules Frente Cerro Galera	Río Aisen	-45.84	-71.78	510	1979-6 / 2016-8	414	10.0	43 (3)
11310003	Chi (1)	Río Blanco Chico Antes Junta Oscuro	Río Aisen	-45.89	-71.71	515	1979-6 / 2016-10	332	28.1	
11310002	Chi (1)	Río Oscuro En Camino Cerro Portezuelo	Río Aisen	-45.91	-71.71	525	1979-6 / 2016-10	387	16.2	
2297	Arg (2)	Río Senguerr - Los Molinos	Rios Senguerr Y Chico	-46.01	-69.51	320	1986-12 / 2013-3	316	0.0	
11504001	Chi (1)	Río Claro Antes Junta Río Ibañez	Río Baker	-46.26	-72.00	217	1985-3 / 1991-5	65	15.6	
11505001	Chi (1)	Río Ibañez En Desembocadura	Río Baker	-46.27	-71.97	220	1970-8 / 2017-6	401	30.9	
11514001	Chi (1)	Río Murta En Desembocadura	Río Baker	-46.43	-72.70	219	1985-3 / 2016-8	350	10.0	
11521001	Chi (1)	Río El Bagno En Chile Chico	Río Baker	-46.55	-71.89	396	1995-12 / 2016-4	229	9.1	
2825	Arg (2)	Río Los Antiguos - Puente Ruta Prov. N43	Lagos Buenos Aires - Pueyrredon	-46.55	-71.64	250	2004-6 / 2016-3	142	0.0	
2824	Arg (2)	Río Deseado - Rp 039	Río Deseado	-46.73	-69.60	225	2004-6 / 2016-3	142	0.0	
11530000	Chi (1)	Río Baker En Desague Lago Bertrand	Río Baker	-47.06	-72.81	200	2003-3 / 2017-6	174	1.7	
11536004	Chi (1)	Río Baker En Angostura Chacabuco	Río Baker	-47.14	-72.73	160	2003-5 / 2017-6	172	1.7	
11536001	Chi (1)	Río Cochrane En Cochrane	Río Baker	-47.26	-72.56	140	1985-6 / 2017-6	288	27.3	
11542001	Chi (1)	Río Baker En Colonia	Río Baker	-47.32	-72.86	105	2001-2 / 2017-6	197	3.0	
11545000	Chi (1)	Río Baker Bajo Ñadis	Río Baker	-47.50	-72.97	45	2003-3 / 2017-6	155	12.4	44 (None)
11711000	Chi (1)	Río Pascua Ante Junta Río Quetru	Río Pascua	-48.16	-73.09	20	2003-3 / 2017-6	169	4.5	
2821	Arg (2)	Río Carreras - Frontera	Río Mayer Y Lago San Martin	-48.21	-72.28	250	1993-2 / 2015-8	264	2.6	
11710000	Chi (1)	Río Pascua En Desague Lago O'higgins	Río Pascua	-48.37	-72.96	260	2003-4 / 2016-8	132	20.5	
11701001	Chi (1)	Río Mayer En Desembocadura	Río Pascua	-48.51	-72.56	350	1985-5 / 2017-6	307	22.7	45 (None)
12125002	Chi (1)	Río Punta Eva En Puerto Eden	Islas Entre Limite Region Y Canal Ancho Y Estrecho De La Concepcion	-49.09	-74.42	10	2004-9 / 2011-11	65	27.0	
2820	Arg (2)	Río De Las Vueltas - Parque Nac. Los Glaciares	Río Santa Cruz	-49.34	-72.86	345	1991-12 / 2015-8	273	4.2	
2827	Arg (2)	Río Shehuen - Chalia - Rn 40	Río Chico	-49.60	-71.38	250	2010-4 / 2016-3	72	0.0	
2801	Arg (2)	Río La Leona - La Leona	Río Santa Cruz	-49.81	-72.05	206	1956-6 / 2016-8	579	19.9	
2802	Arg (2)	Río Santa Cruz - Charles Fuhr	Río Santa Cruz	-50.27	-71.89	206	1955-9 / 2016-8	715	2.3	46 (8)
2815	Arg (2)	Río Centinela - Ruta Prov. N70	Río Santa Cruz	-50.35	-72.51	275	1993-3 / 2013-3	241	0.0	
2822	Arg (2)	Río Mitre - Ruta Prov. N11	Río Santa Cruz	-50.40	-72.73	200	1993-3 / 2016-3	273	1.4	
12280002	Chi (1)	Río Paine En Parque Nacional 2	Costeras Entre Seno Andrew Y R. Hollemberg E Islas Al Oriente	-50.96	-72.79	90	1985-3 / 2017-6	381	4.5	
2828	Arg (2)	Río Vizcachas - Cerro Palique	Río Vizcachas	-50.99	-72.11	255	2010-4 / 2016-3	71	1.4	

12284002	Chi (1)	Río Baguales En Cerro Guido	Costeras Seno Andrew R Hollemberg	-51.01	-72.49	80	1980-8 / 2016-8	428	4.0	47 (None)
12284006	Chi (1)	Río Las Chinas En Cerío Guido	Costeras Entre Seno Andrew Y R. Hollemberg E Islas Al Oriente	-51.02	-72.51	75	1980-6 / 2017-6	440	3.9	
12284003	Chi (1)	Río Vizcachas En Cerro Guido	Costeras Seno Andrew R Hollemberg	-51.03	-72.49	80	1980-9 / 2016-8	415	6.7	
12287001	Chi (1)	Río Grey Antes Junta Serrano	Costeras Seno Andrew R Hollemberg	-51.19	-73.01	50	1981-11 / 2017-6	428	2.9	
12289002	Chi (1)	Río Serrano En Desague Lago Del Toro	Costeras Seno Andrew R Hollemberg	-51.19	-72.96	25	1986-5 / 2017-6	369	4.2	
12289003	Chi (1)	Río Serrano Antes Junta Grey	Costeras Seno Andrew R Hollemberg	-51.20	-72.97	40	1970-5 / 1986-3	113	42.6	
12284004	Chi (1)	Río Las Chinas En Pte Carretero	Costeras Seno Andrew R Hollemberg	-51.22	-72.45	65	1981-11 / 1990-3	101	2.9	
12284007	Chi (1)	Río Las Chinas Antes Desague Del Toro	Costeras Seno Andrew R Hollemberg	-51.25	-72.54	52	1990-3 / 2017-6	329	2.7	
12285003	Chi (1)	Río Tres Pasos En Desague Lago Toro	Costeras Seno Andrew R Hollemberg	-51.28	-72.59	46	2005-1 / 2016-10	142	2.7	
12289001	Chi (1)	Río Serrano En Desembocadura	Costeras Entre Seno Andrew Y R. Hollemberg E Islas Al Oriente	-51.34	-73.11	25	1994-12 / 2017-6	261	6.5	
12285001	Chi (1)	Río Chorrillos Tres Pasos Ruta N 9	Costeras Seno Andrew R Hollemberg	-51.44	-72.48	151	1981-12 / 2016-8	405	5.8	
12291001	Chi (1)	Río Prat En Desembocadura	Costeras Seno Andrew R Hollemberg	-51.56	-72.74	20	2005-1 / 2016-10	142	2.7	
12400003	Chi (1)	Río Tranquilo En Ruta N 9	Costeras E Islas Entre R Hollemberg Golfo Alte. Laguna Blanca	-51.82	-72.16	30	2006-1 / 2016-10	121	9.7	
2818	Arg (2)	Río Gallegos - Puente Blanco	Rios Gallegos Y Chico	-51.89	-71.60	110	1993-2 / 2016-1	275	0.4	
12400004	Chi (1)	Río Hollemberg En Desembocadura	Costeras E Islas R Hollemberg Laguna Blanca	-51.91	-72.43	14	2007-2 / 2016-11	106	12.4	
2819	Arg (2)	Río Penitentes - Rincón De Los Morros	Rios Gallegos Y Chico	-51.92	-71.50	130	1988-2 / 2016-1	283	15.8	
12600001	Chi (1)	Río Rubens En Ruta N 9	Vertiente Del Atlantico	-52.03	-71.95	180	1981-12 / 2017-6	425	3.4	
12660001	Chi (1)	Río Ci-Aike Antes Frontera	Vertiente Del Atlantico	-52.04	-70.06	142	2005-1 / 2016-8	131	9.0	
12622001	Chi (1)	Río Penitente En Morro Chico	Rios Gallegos Y Chico	-52.05	-71.43	170	1980-6 / 2017-6	435	5.0	48 (None)
12452001	Chi (1)	Río Perez En Desembocadura	Costeras E Islas Entre R Hollemberg Golfo Alte. Laguna Blanca	-52.55	-71.97	20	1991-5 / 2017-6	300	7.1	
12802001	Chi (1)	Río Side En Cerro Sombrero	Tierra Del Fuego	-52.77	-69.29	10	1980-6 / 2017-6	437	4.6	
12805001	Chi (1)	Río Oscar En Bahia San Felipe	Tierra Del Fuego	-52.83	-69.77	10	1980-6 / 2016-10	428	4.9	49 (None)
12861001	Chi (1)	Río Cullen En Frontera	Tierra Del Fuego	-52.86	-68.65	67	2005-1 / 2016-8	134	6.9	
12806001	Chi (1)	Río Oro En Bahia San Felipe	Tierra Del Fuego	-52.87	-69.93	10	1980-6 / 2016-3	424	4.3	
12448001	Chi (1)	Río Grande En Isla Riesco	Costeras E Islas Entre R Hollemberg Golfo Alte. Laguna Blanca	-53.00	-71.87	5	1981-12 / 2016-10	326	24.5	
12561001	Chi (1)	Río Grande En Seno Otway	Costeras L Blanca E Magallanes	-53.10	-71.33	22	2007-2 / 2016-7	108	7.7	
12586001	Chi (1)	Río Las Minas En Bt. Sendos	Costeras Entre Lag. Blanca(Inc) Seno Otway Canal Jeronimo Y Magallanes	-53.14	-70.99	185	1982-6 / 2017-6	358	17.3	
12563001	Chi (1)	Río Calete En Seno Otway	Costeras L Blanca E Magallanes	-53.21	-71.62	15	2005-2 / 2016-7	125	12.0	
12585002	Chi (1)	Río Legnadura Antes Bt Sendos	Costeras L Blanca E Magallanes	-53.23	-70.97	49	1982-4 / 1991-12	62	48.3	

12585001	Chi (1)	Río Tres Brazos Antes Bt. Sendos	Costeras L Blanca E Magallanes	-53.28	-70.98	44	1982-6 / 2017-6	388	10.4	
12863002	Chi (1)	Río San Martin En San Sebastian	Tierra Del Fuego	-53.32	-68.64	11	2006-5 / 2016-10	108	16.9	
12865001	Chi (1)	Río Chico En Ruta Y-895	Tierra Del Fuego	-53.55	-68.69	141	2005-1 / 2016-10	129	11.6	
12582001	Chi (1)	Río San Juan En Desembocadura	Costeras Entre Lag. Blanca(Inc) Seno Otway Canal Jeronimo Y Magallanes	-53.65	-70.96	8	1970-5 / 2017-6	470	19.4	50 (6)
12872001	Chi (1)	Río Herminita En Ruta Y-895	Tierra Del Fuego	-53.81	-68.68	107	2005-1 / 2016-2	124	10.1	
12820001	Chi (1)	Río Caleta En Tierra Del Fuego	Tierra Del Fuego	-53.82	-70.17	280	2006-12 / 2017-6	127	2.3	
12876001	Chi (1)	Río Grande En Tierra Del Fuego	Tierra Del Fuego	-53.89	-68.89	100	1981-5 / 2017-6	424	5.1	51 (6)
12878001	Chi (1)	Río Rasmussen En Frontera (Estancia Vicuña)	Tierra Del Fuego	-54.02	-68.62	101	2004-1 / 2016-10	154	3.1	
12825002	Chi (1)	Río Azopardo En Desembocadura	Tierra Del Fuego	-54.50	-68.83	32	2006-2 / 2016-10	126	5.3	
12930001	Chi (1)	Río Robalo En Puerto Williams	Islas Al Sur Del Canal Beagle Y Territorio Antartico	-54.94	-67.65	80	2004-12 / 2017-6	152	1.9	

Supplementary Table 2. Climatic indices used as predictors of Andean streamflow variations in the multivariate regression trials. Data sources: Physical Sciences Division (PSD), Earth System Research Laboratory (ESRL), National Oceanic and Atmospheric Administration (NOAA); Climatic Research Unit, University of East Anglia (CRU); Joint Institute for the Study of the Atmosphere and Ocean, University of Washington (JISAO); Royal Netherlands Meteorological Institute (KNMI); College of Global Change and Earth System Science, China (GCESS), Climate Prediction Center (CPC); National Centers for Environmental Prediction (NCEP); British Antarctic Survey (BAS).

**Climate index description**	**Period**	**Data source**	**Reference**
N34: Mean monthly SST anomalies for the Niño-3.4 region, east-central tropical Pacific (5°N–5°S, 170°–120°W).	1854–present	PSD, ESRL-NOAA https://www.esrl.noaa.gov/psd/	Huang et al. (2017)
SOI (Southern Oscillation Index): Normalized pressure difference between Darwin and Tahiti.	1866-present	CRU http://www.cru.uea.ac.uk/cru/data/soi/	Ropelewski and Jones (1987)
MEIv2 (Multivariate ENSO Index Version 2): Leading Empirical Orthogonal Function of five different variables over the tropical Pacific (30°S-30°N and 100°E-70°W).	1979-present	PSD, ESRL-NOAA https://www.esrl.noaa.gov/psd/	Wolter and Timlin (2011)
PDO (Pacific Decadal Oscillation): Leading PC of monthly SST anomalies in the North Pacific Ocean, poleward of 20°N.	1900–present	JISAO http://jisao.washington.edu/pdo/	Mantua et al. (1997)
AMO (Atlantic Multi-decadal Oscillation): Mean monthly Atlantic SST anomalies over 0°-60°N, 0°-80°W, minus SST averaged over 60°S-60°N.	1854–present	KNMI and NOAA https://climexp.knmi.nl/	Trenberth and Shea (2006)
TSA (Tropical Southern Atlantic Index): Mean monthly SST anomalies from the equator to 20°S, and 10°E to 30°W.	1948–present	PSD, ESRL-NOAA https://www.esrl.noaa.gov/psd/	Enfield et al. (1999)
SAODI (South Atlantic Ocean Dipole Index): Difference of SST anomalies for the regions 10ºE–20ºW, 0º–15ºS and 10º–40ºW, 25ºS – 40ºS in the Atlantic Ocean.	1854-present	GCESS http://ljp.gcess.cn/dct/page/65544	Nnamchi et al. (2011)
AAO (Antarctic Oscillation): Leading PC of 850-hPa geopotential height anomalies south of 20°S.	1979–present	CPC, NOAA–NCEP http://www.cpc.ncep.noaa.gov/data/indices/	Mo (2000)
SAM (Southern Annular Mode): Normalized difference of zonal pressures between ca. 40° and 65°S measured at a fixed number of stations.	1957-present	BAS http://www.nerc-bas.ac.uk/icd/gjma/sam.html	Marshall (2003)

Supplementary References

Enfield, D.B., A.M. Mestas, D.A. Mayer, and L. Cid-Serrano, 1999: How ubiquitous is the dipole relationship in tropical Atlantic sea surface temperatures? JGR-Oceans, 104, 7841-7848.

Huang, B., P.W. Thorne, V.F. Banzon, T. Boyer, G. Chepurin, J.H. Lawrimore, M.J. Menne, T.M. Smith, R.S. Vose, and H. Zhang, 2017: Extended Reconstructed Sea Surface Temperature, Version 5 (ERSSTv5): Upgrades, Validations, and Intercomparisons. J. Climate, 30, 8179–8205.

Mantua, N.J. and S.R. Hare, Y. Zhang, J.M. Wallace, and R.C. Francis, 1997: A Pacific interdecadal climate oscillation with impacts on salmon production. BAMS, 78, 1069-1079.

Marshall, G. J., 2003: Trends in the Southern Annular Mode from observations and reanalyses. J. Clim., 16, 4134-4143.

Mo, K. C., 2000: Relationships between Low-Frequency Variability in the Southern Hemisphere and Sea Surface Temperature Anomalies. J. Climate, 13, 3599-3610.

Nnamchi, H.C., J. P. Li. and R. N. C. Anyadike, 2011: Does a dipole mode really exist in the South Atlantic Ocean? J. Geophys. Res., 116, doi: 10.1029/2010JD015579.

Ropelewski, C.F. and Jones, P.D., 1987: An extension of the Tahiti-Darwin Southern Oscillation Index. Monthly Weather Review 115, 2161-2165.

Trenberth, K. E., and Shea, D. J. (2006), Atlantic hurricanes and natural variability in 2005, Geophys. Res. Lett., 33, L12704, doi:10.1029/2006GL026894.

Wolter, K., and M. S. Timlin, 2011: El Niño/Southern Oscillation behaviour since 1871 as diagnosed in an extended multivariate ENSO index (MEI.ext). Intl. J. Climatology, 31, 1074-1087.
